# Exploring oak processionary caterpillar induced lepidopterism (Part 1): unveiling molecular insights through transcriptomics and proteomics

**DOI:** 10.1007/s00018-024-05330-z

**Published:** 2024-07-27

**Authors:** Andrea Seldeslachts, Marius F. Maurstad, Jan Philip Øyen, Eivind Andreas Baste Undheim, Steve Peigneur, Jan Tytgat

**Affiliations:** 1 Toxicology and Pharmacology, Department Pharmaceutical and Pharmacological Sciences, KU Leuven , Leuven, Vlaams-Brabant, Belgium; 2 Centre for Ecological and Evolutionary Synthesis, Department of Biosciences, University of Oslo, Oslo, Norway; 3 Present Address: Division of biotechnology and plant health & viruses, bacteria and nematodes in forestry, agriculture and horticulture, Norwegian Institute of Bioeconomy Research (NIBIO), Oslo, Norway

**Keywords:** Caterpillar venom, *Thaumetopoea processionea*, Irritant contact-dermatitis, Transcriptomics and proteomics

## Abstract

Lepidopterism, a skin inflammation condition caused by direct or airborne exposure to irritating hairs (setae) from processionary caterpillars, is becoming a significant public health concern. Recent outbreaks of the oak processionary caterpillar (*Thaumetopoea processionea*) have caused noteworthy health and economic consequences, with a rising frequency expected in the future, exacerbated by global warming promoting the survival of the caterpillar. Current medical treatments focus on symptom relief due to the lack of an effective therapy. While the source is known, understanding the precise causes of symptoms remain incomplete understood. In this study, we employed an advanced method to extract venom from the setae and identify the venom components through high-quality *de novo* transcriptomics, venom proteomics, and bioinformatic analysis. A total of 171 venom components were identified, including allergens, odorant binding proteins, small peptides, enzymes, enzyme inhibitors, and chitin biosynthesis products, potentially responsible for inflammatory and allergic reactions. This work presents the first comprehensive proteotranscriptomic database of *T. processionea*, contributing to understanding the complexity of lepidopterism. Furthermore, these findings hold promise for advancing therapeutic approaches to mitigate the global health impact of *T. processionea* and related caterpillars.

**Supplementary Information:**

The online version contains supplementary material available at 10.1007/s00018-024-05330-z.

## Introduction

Recent outbreaks of the oak processionary caterpillar (*Thaumetopoea processionea*) have reached epidemic levels, leading to significant health and economic consequences [1, 2]. The situation is expected to rise in the future as the effects of global warming continue to promote caterpillar survival and facilitate their range expansion [3]. From the third life stage, *T. processionea* is equipped with up to 1 million harpoon-shaped setae that are sharp enough to cause mechanical injury and penetrate the skin and mucosa [4]. Unlike other insects such as wasps and bees, the setae lack specialized gland cells for venom production, rendering the characterization of the exact mechanism of venom synthesis elusive up to the present day [5]. Often, accidental contact involves a single caterpillar or, more severely a colony with a dangerous number of caterpillars [1]. However, the reactions are also seen over a considerable distance from the origin. As part of their natural defense system against predators, *T. processionea* releases the setae into the air and the environment, where they remain active for approximately 10 years [6, 7]. The fact that the setae remain active for many years suggests that the venom is composed of highly stable and robust molecules well-protected by the outer casing and likely the foundation for causing the symptoms. Direct or airborne contact is enough to break the setae and release a mixture of bio-active molecules to exert local and eventually systemic pathological effects that only appear 6 to 8 h after the contact [6, 7]. Symptoms of contact with setae in humans and animals (pets, horses, grazers) include local itch, redness, breathing problems, conjunctivitis, swallowing problems, and may escalate to a life-threatening shock [8, 9]. Even in the 21st century, treatment of the envenomation by these caterpillars is still clinically challenging and mainly symptomatic and supportive [1]. Until today, no effective medical treatment is available on the market.

Although the source is obvious, many aspects of what causes the symptoms are yet to be elucidated. Contrasting with multiple biochemical and pharmacological studies on the content and interaction of venom components of snakes, spiders, and scorpions, the literature on caterpillar venoms remains scarce. Caterpillar species are still regarded as a neglected group of venomous animals, and consequently, little is known about the protein/peptide content in the venom. A few studies have been conducted on the proteins of the urticating setae from species in the same genus, *Thaumetopoea pityocampa* [10–13] and *Ochrogaster lunifer* [14]. In these studies, specific attention is directed towards the three documented allergens: Thaumetopoein, Tha p 1, and Tha p 2. Thaumetopoein, a heterodimer consisting of a 13-kDa and 15-kDa subunit, was the first protein isolated from the setae of *T. pityocampa*, exhibiting a non-IgE dependent effect on mast cells [8, 13, 15, 16]. Despite being the first specific protein isolated, Thaumetopoein has never been sequenced, and no further homology or function has been associated with it. Subsequently, Tha p 1 and Tha p 2, were identified and acknowledged by the World Health Organization and International Union of Immunological Societies as major caterpillar allergens due to their IgE-binding properties in sensitized patients [12, 17]. While the biological function of these allergens remains unknown, structural information has been documented. Tha p 1 was categorized as an odorant binding protein with a molecular weight of 15 kDa [12, 18]. In contrast, Tha p 2, unrelated to Tha p 1, has a molecular weight of 13 kDa and is present in all Notodontidae species investigated in the study by *Berardi et al. (2015)* including *T. processionea* [8, 10, 11, 19]. Besides Tha p 2, no further information is known about the venom composition of *T. processionea* and how it can be compared to the previous described *Thaumetopoea* species.

To the best of our knowledge, no sequenced transcriptome or complete proteomic data is available for *T. processionea*, which significantly hampers the understanding of *T. processionea* envenomation. Unraveling the venom composition marks a significant stride, enhancing the current state-of-the-art and opening avenues for the development of more efficient treatment alternatives.

## Materials and methods

### Collection and venom extraction from setae of *T. processionea*

Larval colonies of *T. processionea* were collected in June and July 2020 from oak (*Quercus*) trees in Mol (Limburg, Belgium). Each larva of L5-L6 instar was taken out from the tent in a biosafety cabinet and put in a falcon tube. The setae were collected by freezing the larvae with liquid nitrogen at -196 °C and shaking to release the setae. Approximately 200,000,000 urticating setae were collected from 200 larvae and stored at -80 °C. The setae were dissolved in 50% acetonitrile (ACN) solution, stirred for 4 h with a magnetic stirrer, and crushed by sonification at 30.0 Hz for 15 min. The solution was clarified through centrifugation at 12,500 rpm for 10 min using an Eppendorf centrifuge 5810R (Eppendorf AG, Germany). The resulting supernatant was subjected to lyophilization. The desiccated substance obtained through this process was employed for subsequent analytical investigations.

### Transcriptomic analyses

One late-instar caterpillar was dissolved in RNA*later*™ Solution (Thermo Fisher Scientific, Vilnius, Lithuania), flash-frozen with liquid nitrogen and kept at -80 °C for RNA extraction. Before RNA extraction, the head and the gut were removed with a lab knife, followed by RNA extraction with a Qiagen RNeasy Micro kit (Qiagen, USA). Total RNA concentration and purity were assessed using a spectrophotometer at absorbance 260 nm and 280 nm (NanoDrop ND-1000 UV/Vis, Delaware, USA), and the integrity was checked using an Agilent 2100 Bioanalyzer (Agilent, USA). The extracted RNA was then sequenced by a combination of long- and short-read methods: using Single Molecule Real-Time (SMRT) Iso-Seq (Pacific Biosciences, United Kingdom) to obtain highly accurate long-reads and an Illumina NovaSeq to obtain highly accurate short paired end reads. For both methods, ≥ 300 ng of high-quality total RNA was used (A260/280 ratio ∼ 2.0; A260/230 ratio ≥ 2.0; RNA integrity number ≥ 8.0).

For the PacBio Iso-Seq, the library was prepared using the Pacific Biosciences protocol for Iso-Seq™ Express Template Preparation for Sequel II Systems. The library was sequenced on a Pacific Sequel II instrument using Sequel II Binding kit 2.0 and Sequencing Chemistry v2.0. Sequencing was performed on one SMRT cell. The resulting raw subread data were further polished using SMRT Link v9.0 to generate circular consensus sequences (CCS) from the raw subreads. Only CCS reads with a phred score of ≥ Q20 were used further. These high-fidelity reads (HiFi reads = CCS Reads with predicted accuracy ≥ Q20) were classified in full-length non-chimera (FLNC) transcript reads depending on the presence of 5’ cDNA primer sequence, 3’ cDNA primer sequence, and polyA tail sequence. Next, FLNC reads were clustered in the same isoform if there is < 100 bp difference at the 5’ start, < 30 bp difference at the 3’ end, and < 10 bp in the internal gap. In the last step, the isoforms were classified into high-quality (HQ) and low-quality (LQ) isoforms based on the accuracy value (HQ > 0,99). Library preparation, sequencing, HiFi read generation, and IsoSeq processing was done at the Norwegian Sequencing Centre PacBio node, University of Oslo Department of Biosciences, Oslo, Norway.

For the Illumina NovaSeq platform, TruSeq libraries featuring 150 bp inserts were prepared and paired-end sequenced following the manufacturer’s guidelines. Library and sequencing were performed at the Norwegian Sequencing Centre Illumina node at the Oslo University Hospital, Oslo, Norway. The reads were subsequently subjected to FastQC v11.9 [20] to check quality and length distribution. The adapter sequences (TruSeq3-PE-adapters) and low-quality reads were trimmed with Trimmomatic v0.39 [21]. Quality trimming was carried out using a window-based approach with a sliding window of 4 bases and a minimum quality threshold of 20. Sequences shorter than 60 bases were excluded after trimming.

Based on the polished reads, three transcriptomes were generated: (1) IsoSeq library, (2) *de novo* assembly of short-reads generated using default settings in Trinity v2.10 [22], and (3) short-reads assembled with the long-reads as reference with Trinity v2.10. The overlap and global completeness of each assembly were assessed with CD-HIT v4.8.1 [23] and BUSCO v5.0.0 [24], respectively, using universal single-copy orthologs from Lepidoptera as our database for the BUSCO analyses. To assess the relative abundance of each transcript, the paired trimmed reads were mapped back to the transcriptome assembly using bowtie2 [25]. The abundance values were calculated as Transcripts Per Million (TPM) from the mapped reads using RSEM v1.3.3 [26]. TransDecoder v5.5.0 [27] was used to extract the open reading frames (ORF) encoding 50 amino acids or more in order to build an amino acid sequence database for automated searching of the MS data obtained by proteomics. To ensure that no small peptides were overlooked, we repeated the search using a database that included all previously identified amino acid sequences as well as predicted open reading frames (ORFs) ranging from 25 to 50 amino acids in length. The accuracy and completeness of the toxin-encoding contigs in each assembly were examined by the mapped read data and comparison of translated open reading frames with proteomics data. The assembly with the highest quality was chosen based on global completeness and toxin accuracy.

### Pipeline analysis SRA data from *T. pityocampa*

Available data for *T. pityocampa* was downloaded from SRA (Sequence Read Archive) and converted using SRA-Toolkit v3.0.3 (SRR12643372, SRR12643373, SRR12643374, SRR12643375, SRR12643376, SRR12643377, SRR12629075, SRR12629076, SRR12629077, SRR12629078, SRR12629079, SRR12629080, SRR12629081, SRR12627525, SRR12627526, SRR12627527, SRR12627528, SRR12627529, SRR12627530, SRR12627531, SRR12627532, SRR6706317, SRR6706318, SRR6706320, SRR6706321, SRR6706221, SRR6706220, SRR6706222, SRR6706223, SRR6706225, SRR6511320, SRR6511321, SRR6511322, SRR6511323, SRR6511324). Trimommatic v0.39 [21] was used for adapter removal and quality trimming of reads using a sliding window of 4 bases, a minimum quality threshold of 30 and sequences shorter than 80 bases were excluded after trimming. Trimmed reads were assembled using Trinity v2.15.1 [22] and TransDecoder 5.7.0 [28] was used to predict ORFs with a minimum amino acid length of 50.

### Proteomic characterization of *T. processionea* urticating setae

The proteomic data was obtained by analyzing the extracted venom via a top-down and bottom-up approach with trapped ion mobility spectrometry combined with a time-of-flight mass spectrometer (timsTOF Pro, Bruker, USA). For these timsTOF analyses, three different samples were prepared: native (top-down), reduced and alkylated (RA) (top-down), and RA digested (RAT) (bottom-up). In this context, the analysis of untreated venom offers insights into the original toxin masses and fragmentation patterns, especially for peptides that do not contain disulfide bonds [29]. On the other hand, the RA-treated sample enhances fragmentation and provides details about the cysteine content of these peptides [29]. Additionally, RA digested samples yield information about components that would have been too large to analyze [29].

Native samples were dissolved in a solution containing 5% acetonitrile (ACN) and 1% formic acid. The reduced and alkylated samples were reduced with 5 mM dithiothreitol in 50 mM ammonium bicarbonate and 5% ACN for 5 min at 65 °C and alkylated with 10 mM iodoacetamine in 50 mM ammonium bicarbonate and 5% ACN for 30 min at 30 °C. The RA digested samples were, following reduction and alkylation, digested overnight with trypsin (42 ng/µL in 50 mM ammonium bicarbonate and 5% ACN at 37 °C). Upon complete digestion, 1% formic acid was added to inactivate trypsin. The three samples were desalted using a C18 ZipTip (Thermo Fisher, USA). After desalting, samples were vacuum centrifuged for 20 min and dissolved again in 1% formic acid for further analysis on a timsTOF Pro mass spectrometer (Bruker, USA). The datasets acquired from the venom samples were categorized as untreated (N), subjected to reduction and alkylation (RA), or treated with reduction, alkylation, and trypsin digestion (RAT).

The resulting mass spectrometry datasets were searched against the translated predicted ORFs from the transcriptome and a list of 23 common contaminants via Peaks Studio v8.5. Peaks Studio estimates the identification of false positives using the decoy-based false discovery rate (FDR). Only peptide/protein identification with a corresponding local FDR of < 0.1% was considered significant. Moreover, peptide matches were generated with a minimum − 10lgP score of 15 (*p* ≤ 0.03). The -10lgP value is related to the probability (*p*-value) that a peptide-spectrum match is due to random chance ($p= {10}^{- \frac{score}{10}}$) and thus a false positive. To ensure robust protein identification, the % coverage was also manually inspected. The criteria were set that at least two peptides or one peptide with more than 10% coverage are required in order to provide confidence in the reliability of the identified proteins in the venom of *T. processionea*. In the search, depending on the sample preparation (N, RA, RAT), specific parameters were set: Trypsin as cleavage enzyme allowed up to two missed cleavage, parent mass error tolerance of 15 parts per million, and fragment mass error tolerance of 0.03 Da. Cysteine carbamidomethylation and methionine oxidation as a fixed modification.

### Classification and annotation of the *T. processionea* urticating setae proteome

The vast majority of polypeptide toxin precursors are encoded as prepropeptides that begin with a signal peptide sequence. We therefore used SignalP v4.0 [30] to detect the signal sequence, and excluded proteins and peptides without a predicted signal peptide region from the list of toxin candidates for further analysis and classification. The resulting list of putative venom components were then classified and functionally annotated using InterProScan v5.47 [31] and Blastp searches against UniProtKB (downloaded 29th September 2021; [32]) with BLAST v2.10.1 [33]. Sequences lacking identifiable functional or structural motifs are designated as ‘unknown’. The amino acid sequences of each toxin type were aligned with MAFFT v7 using L-INS-i [34] to look at several domains and compare which are present or absent.

### Phylogenetic analysis

To explore the complete molecular diversity, we also performed Blastp searches of sequences from the extracted venom against the translated transcriptome with an E-value set to 1e-2. The amino acid sequences were aligned with MAFFT v7 using L-INS-i [34]. For the phylogenetic analysis, IQTree v1.5.5 [35] and Archaeopteryx 0.9928 beta were used to reconstruct the molecular phylogenies by maximum likelihood methods and estimated branch support values by ultrafast bootstrap. For the TPTX_1_ family analysis, the phylogenetic tree was constructed under the maximum likelihood VT + I + G4 model. This model was selected using Bayesian Information Criterion (BIC) to ensure the best fit for the data. For the Kazal family, the alignments were performed with multi-domain peptides, which were split into their individual domains to ensure accurate alignment and tree construction. The tree was calculated by maximum likelihood under the WAG + F + I + G4 model, which was also chosen according to BIC.

### Predicted molecular modeling via AlphaFold2

To gain further insight into the structural diversity of identified venom components we modelled their 3D structure using AlphaFold2 [36]. All 3D structures were predicted using the MMseqs2 application interface in ColabFold, implementing the AlphaFold2 source code. The sequences of venom components were also queried against the RCSB Protein Data Bank (PDB) website to identify analogous structures. Graphical visualization of the AlphaFold2 modeled structures was conducted using PyMol v2.5 (Schrödinger, LLC). Besides visualization, PyMol v2.5 was also employed for the alignment of acquired 3D structures. The alignment was performed with the align function of PyMol using the predicted mature sequence. The Root Mean Square Deviation (RMSD) was used to evaluate the alignment between the two structures. The lower the RMSD value, the closer the alignment.

## Results

### Harmonizing short- and long-read sequencing data to generate the *T. processionea* transcriptome

To gain insight into the venom repertoire of *T. processionea*, we used a combination of short- and high-accuracy long-read sequencing technologies to obtain the transcriptome of *T. processionea*. The details of the obtained raw reads and further polished data obtained from PacBio IsoSeq and Illumina NovaSeq are summarized in Table 1. We obtained 3,838,782 raw PacBio reads that were processed through the standard SMRT Link Analysis pipelines to yield 552,459 HiFi reads. These reads comprised 548,307 FLNC reads that were clustered by the IsoSeq pipeline to give 14,858 high-quality transcript isoforms with an average read length of 2492 bp. The complete IsoSeq-based transcriptome had a BUSCO score of 60.9% (28.7% single, 32.2% duplicate, 2.0% fragmented, and 37.1% missing), suggesting an unexpected incompleteness.

**Table 1:** Details encompassing raw data generated through RNA sequencing using PacBio IsoSeq and Illumina NovaSeq, as well as the combined dataset, the assembly methodology, processed data, and the results of the BUSCO analysis

Sequencing method	Raw data	Assembly method	Processed data	BUSCO
PacBio Long-read SMRT Iso-Seq	3,838,782 reads	SMRT Link analysis	*CCS or *HiFi: 552,459 reads *FLNC: 548,307 reads High-quality (*Hq): 14,858 transcripts Read length average: 2492 bp	CCS: 60.9%
Short-read Illumina NovaSeq	41,095,405 paired end reads after Trimmomatic	Trinity de novo	Trinity assembly: 108,951 contigs	71.4%
Combined long- and short-reads	41,095,405 paired end reads obtained after Trimmomatic combined with 552,459 HiFi reads as reference	Trinity with Long-read reference data	Trinity assembly: 109,866 contigs	71.6%

*CCS: Circular Consensus Sequences

*HiFi: High-Fidelity reads (CCS Reads with predicted accuracy ≥ Q20)

*FLNC: Full-Length Non-Chimeric reads

*Hq: High-quality

*SMRT: Single Molecule Real-Time

We obtained 56,852,238 paired end Illumina reads, which after processing with Trimmomatic yielded 41,095,405 high-quality paired reads. The resulting dataset was assembled with Trinity to generate both a *de novo* assembly and a reference-guided assembly with our HiFi read data as a reference. The *de novo* assembly produced 108,951 contigs with a BUSCO score of 71.4% (45.7% single, 25.7% duplicate, 6.5% fragmented and 22.1% missing), while the reference-guided assembly produced 109,866 contigs and had a BUSCO score of 71.6% (56.3% single, 15.3% duplicate, 6.3% fragmented, and 22.1% missing).

Considering these detailed BUSCO results, the IsoSeq-based transcriptome demonstrates a lower overall completeness compared to the other two. The Illumina-based transcriptome shows an improvement in overall completeness compared to the IsoSeq-based transcriptome. The higher percentage of complete and single-copy BUSCOs (45.7%) indicates better gene representation, albeit with some redundancy. The reference-guided assembly achieves the highest completeness with a 56.3% percentage of complete and single-copy BUSCOs suggesting a more accurate representation of the transcriptome and thus a favorable choice for further downstream analyses.

Subsequently, TransDecoder was utilized to extract ORFs, and the CD-HIT package was applied to eliminate redundant sequences. This process yielded a total of 177,176 contigs encoding amino acid sequences, representing the transcriptome. As the genome and transcriptome of *T. processionea* were previously unavailable, this newly established high-quality transcriptome can now identify, for the first time, the coding regions of proteins in *T. processionea*.

### Exploring venom diversity: identification and classification of *T. processionea* toxins through integrated transcriptomic and proteomic analyses with bioinformatic tools

**Fig. 1:**
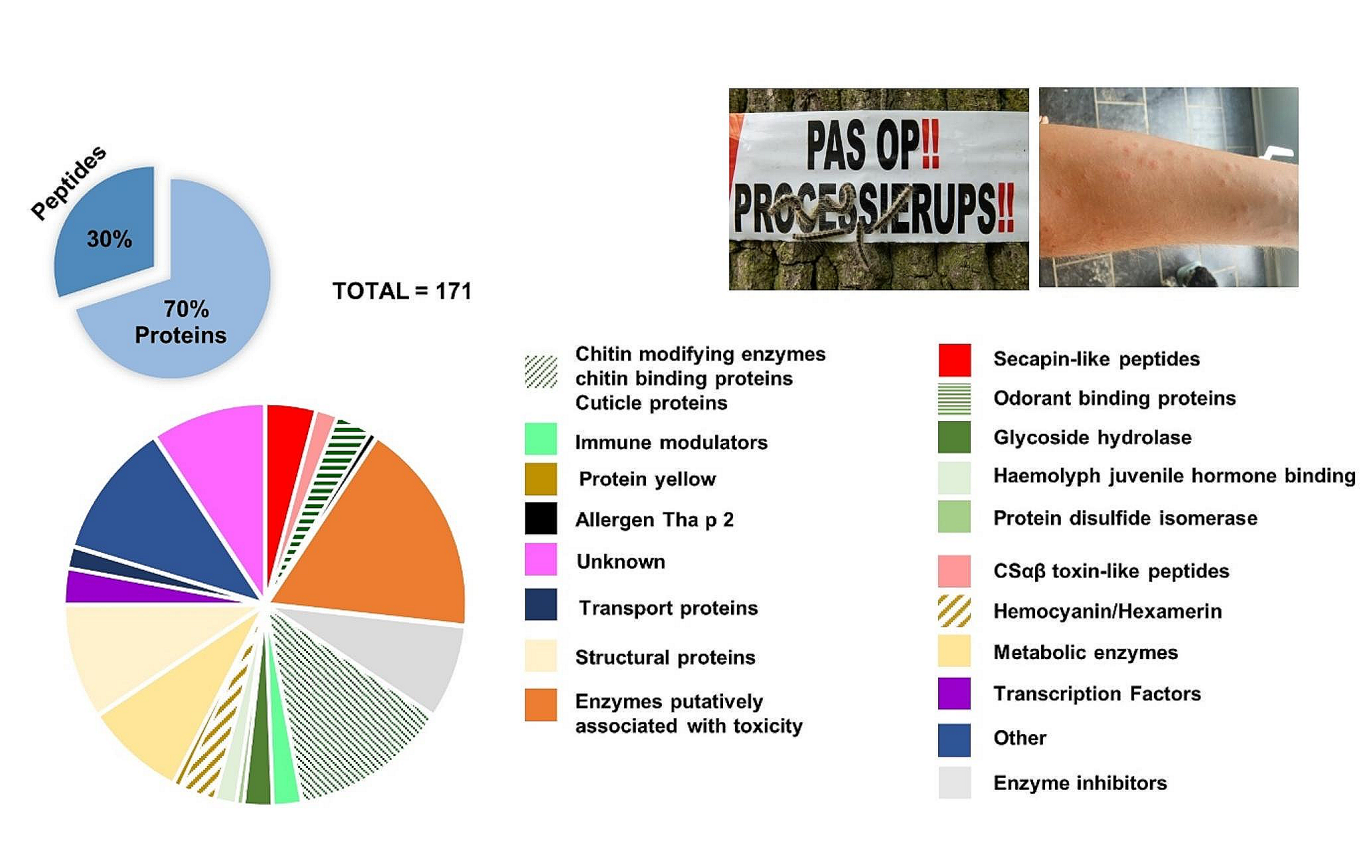
Identification and classification of *Thaumetopoea processionea* toxins. 19 categories were identified in *T. processionea* venom by mapping the proteome (mass spectra generated by timsTOF mass spectrometer) to the transcriptome (obtained from combined short-read Illumina NovaSeq sequencing and long-read PacBio IsoSeq sequencing). SignalP, InterProScan and UniProt Blastp annotation were employed to assign the toxin candidate sequences to the specific categories. The proportions displayed represent the number of peptides/proteins within each category divided by the total number of 171 components, providing a percentage of each category within the total venom composition

To characterize the toxin candidates expressed in the venom extract of *T. processionea*, the reference-guided assembly was translated with Transdecoder to yield 177,176 ORFs, which were used as a protein database to search venom LC-MS/MS data from timsTOF Pro. This search yielded a total of 648 ORFs, of which 171 contained a signal peptide sequence. It is widely recognized that secreted venom components are characterized by a signal peptide signature. This signal peptide signature is a short amino acid sequence at the N-terminus that guides the protein/peptide to the endoplasmic reticulum [37]. Its presence suggests that the venom proteins/peptides are transported through a secretory pathway to reach their final destination [38]. These 171 venom components were divided into 19 categories (Fig. 1). In these categories we identified 23 families that may contribute to the setae toxicity based on (1) sequence homology, (2) cysteine pattern similarity, (3) conserved domains, or (4) common BLAST hits. To establish a consistent nomenclature, peptides/proteins from similar families were designated with the prefixes TPTX (Thaumetopoeinae toxin) with a subscript that indicates the putative family followed by Tp that stands for *T. processionea* and the numerical identifier at the end is unique [39]. The prefix ‘U’ at the beginning stands for unknown pharmacological activity. A complete list of the 171 proteins and peptides, including predicted signal peptides, category, family, and predicted domains, along with their putative biological functions identified by UniProt and proteins identified by Blastp, is provided in Supplementary Table S1. The results of the RSEM analysis are presented in Supplementary Figure S1. It should be noted that the transcript abundances are of indicative nature given that it is not known when or where the toxins are made.

Among 171 venom components, 70% of them are proteins. Within the remaining 30%, categorized as peptides (sequences < 100 amino acids in length), the presence of cysteine residues characterizes half of the sequences. Of these, 18 peptides have an even number of cysteine residues and 7 exhibit an odd number of cysteines. Peptides possessing an odd number of cysteines have the potential to form dimers or associate with other proteins [40]. However, whether this is the case for the peptides reported here is yet to be substantiated.

### Venom chronicles: exploring the 23 putative toxic families in *T. processionea*

#### Secapin-like peptides

**Fig. 2:**
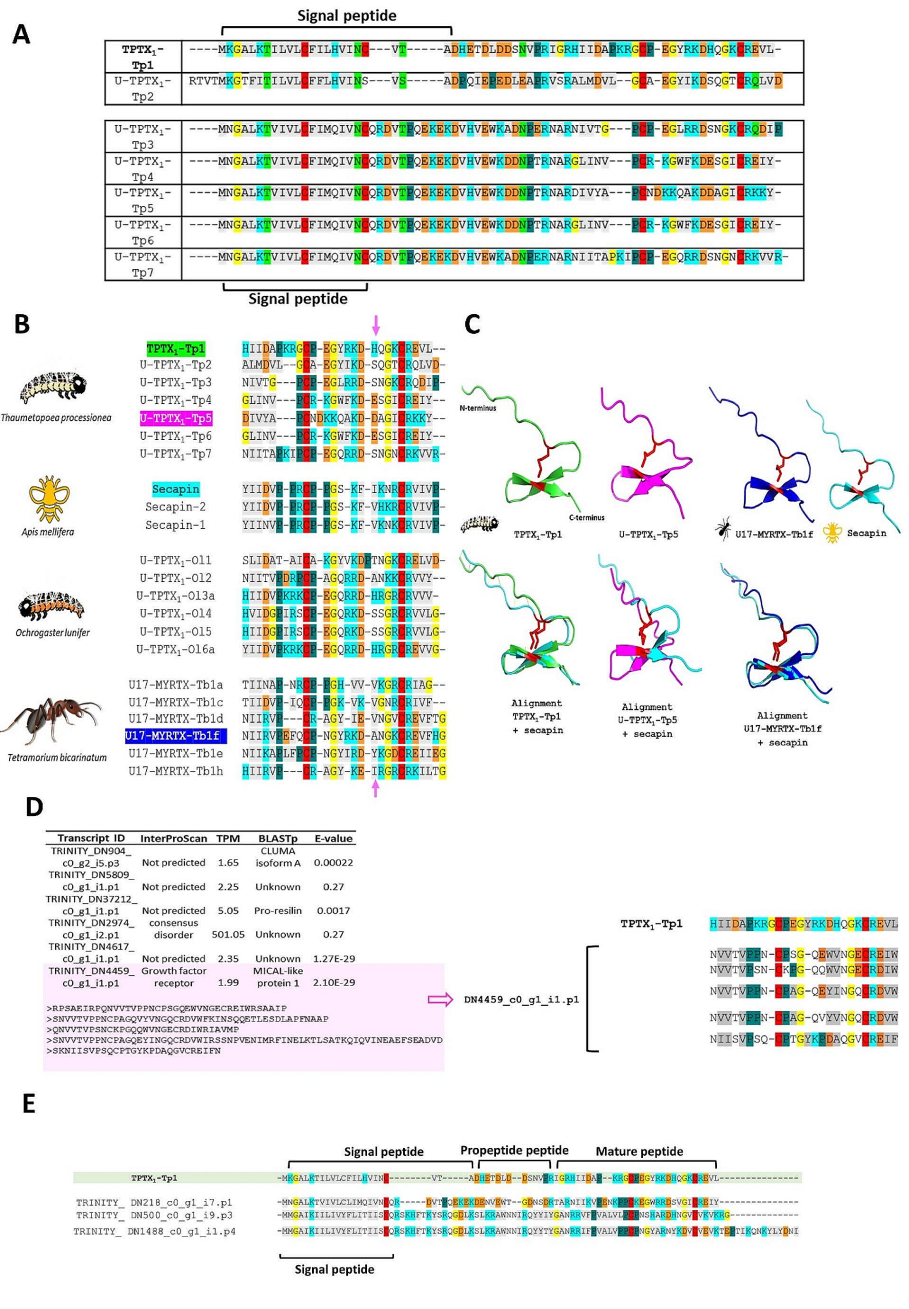
Secapin-like peptide family in the venom of *Thaumetopoea processionea*. (**A**) Alignment of *T. processionea* secapin-like peptides with signal *P*-sequence. (**B**) Alignment of *T. processsionea* peptides with secapins from *Apis mellifera*, secapin-like peptides from *Ochrogaster lunifer* and *Tetramorium bicarinatum*. The pink arrow indicates a residue that has previously been identified as undergoing positive selection [41]. (**C**) AlphaFold2 predicted 3D structure of TPTX_1_-Tp1 and U-TPTX_1_-Tp5 from *T. processionea* compared with a secapin-like peptide U17-MYRTX-Tb1f from *Tetramorium bicarinatum* and compared and aligned with secapin from *Apis mellifera*. (**D**) Blast search of TPTX_1_-Tp1 against the assembled transcriptome of *T. processionea*. (**E**) BLAST analysis on TPTX_1_-Tp1 against a self-assembled transcriptome of *Thaumetopoea pityocampa*, generated using Trinity and based on available Sequence Read Archive (SRA) data. Residues in gray represent amino acids with a hydrophobic side chain, residues in light blue represent a positively charged chain, negatively charged side chain in orange, glycine in yellow, proline in green, cysteine in red and amino acid with an uncharged polar side chain do not have a color

We identified seven secapin-like peptides ((U)-TPTX_1_-Tp(1–7)) that all share a common cysteine scaffold (Fig. 2A). Confirmation of their presence in the venom of *T. processionea* was achieved through the proteotranscriptomic pipeline, revealing high -10lgP scores. For example, TPTX_1_-Tp1 exhibited a -10lgP score of 260.68, and U-TPTX_1_-Tp5 showed a -10lgP score of 214.36. A Blastp search revealed that TPTX_1_-Tp1 shares 81.2% similarity with U17 myrmicitoxins, which are identified in ant venom and classified as secapin-like peptides. Aligning (U)-TPTX_1_-Tp(1–7) found in *T. processionea* venom with secapins from *Apis mellifera*, secapin-like peptides from *Ochrogaster lunifer*, and myrmicitoxins from *Tetramorium bicarinatum* revealed shared features suggesting a common ancestor. These include conserved residues and a highly similar structural fold characterized by a single disulfide bridge that likely plays a crucial role in sustaining a high degree of structural stability (Fig. 2B). In this context, a pair of amino acids with hydrophobic side chains resemble the N-terminus (grey), and an arginine-lysine-rich (light blue) region is visible around the second cysteine. Around the first cysteine, almost all sequences have a proline (green) and a conserved glycine (yellow) residue. From the overall domain architecture, we can tell that most molecules are highly charged. For example, TPTX_1_-Tp1 has 3 arginine residues and 2 lysine residues, while U-TPTX_1_-Tp5 has 1 arginine residue and 5 lysine residues. Moreover, we want to highlight the residues indicated with a pink arrow on the top and bottom of the alignment. For TPTX_1_-Tp1, a positive charge, and for U-TPTX_1_-Tp5, a negative charge was visible, while secapin and U17-MYRTX-Tb1f both have a hydrophobic residue that has previously been found to be under positive selection [41]. According to Barassé et al. (2022), a variation in charge and polarity on this specific position could potentially influence the conformation and functionality of the peptides [41]. In addition, (U)-TPTX_1_-Tp(1–7) lack the amidation tag, characterized by the absence of the glycine (G) or the basic residues lysine-arginine (KR) or lysine (K). These findings suggest that the peptides don’t possess the necessary features for C-terminal amidation in contrast with the peptides found in the venom of *O. lunifer* and *T. bicarinatum* [14, 41].

To further compare the structural similarities of venom secapins, the 3D structures of TPTX_1_-Tp1 and U-TPTX_1_-Tp5 were predicted using AlphaFold2 (Fig. 2C). In these 3D structures, the key common structural feature is a double-stranded antiparallel β-sheet connected by a β-turn, also known as a β-hairpin motif. Both the N-terminus and C-terminus exhibit an unstructured tail. The same 3D structure was also described for U17-MYRTX-Tb1a and U-TPTX_1_-Ol1 [14, 41]. Furthermore, the RMSD value of 2.342 Å, calculated for 137 aligned atoms, indicates a relatively high level of structural similarity between the predicted mature molecular structures of TPTX_1_-Tp1 from *T. processionea* and secapin from *A. mellifera*. For U-TPTX_1_-Tp5 from *T. processionea* and secapin from *A. mellifera*, a close match between the atoms in the mature structures was obtained (RMSD of 0.136 Å). These findings point to a remarkable degree of similarity and consistency between (U)-TPTX_1_-Tp(1–7), U17-MYRTX-Tb, U-TPTX_1_-Ol, and secapin.

To identify additional paralogs of secapin-like sequences in the transcriptome of *T. processionea*, we performed a Blast search of the sequence of TPTX_1_-Tp1 against our reference-guided assembly, revealing a total of 12 sequences (with an E-value set to 1e-2) (Fig. 2D) including the secapin-like peptides found in the venom (Fig. 2A). We also conducted a Blast analysis on TPTX_1_-Tp1 against a transcriptome assembly of *T. pityocampa*, generated using Trinity based on available SRA data. With an E-value set to 1e-2, we identified 7 peptides similar to TPTX_1_-Tp1 (Fig. 2E). TRINITY_ DN218_c0_g1_i7.p1 showed a 42% similarity to TPTX_1_-Tp1 and several conserved residues. TRINITY_DN500_c0_g1_i9.p3 and TRINITY_DN1488_c0_g1_i3.p4 also preserved some residues but had an extended C-terminus. Additionally, half of the transcripts identified from *T. processionea* were absent from the extracted venom. However, there is currently no evidence suggesting their role as toxins. Among these likely non-toxin paralogs, one sequence contained distinct repeat domains as well as a growth factor receptor domain. A Blast search of TPTX_1_-Tp1 against UniProtKB yielded diverse hits from hymenoptera (bees and wasps) and lepidoptera. These hits included short or single domain matches as well as hits to larger proteins and growth factor domains. While this hints at potential conservation across stinging hymenoptera and certain insect species, it also suggests the possibility of convergent evolution. Phylogenetic analysis of the secapin-like sequences from *Thaumatopoea* and *Ochrogaster* was not particularly informative (Supplementary Fig. S2), and further analysis is needed to conclusively determine the evolutionary origin of TPTX_1_-Tp1.

#### CSαβ toxin-like peptides

**Fig. 3:**
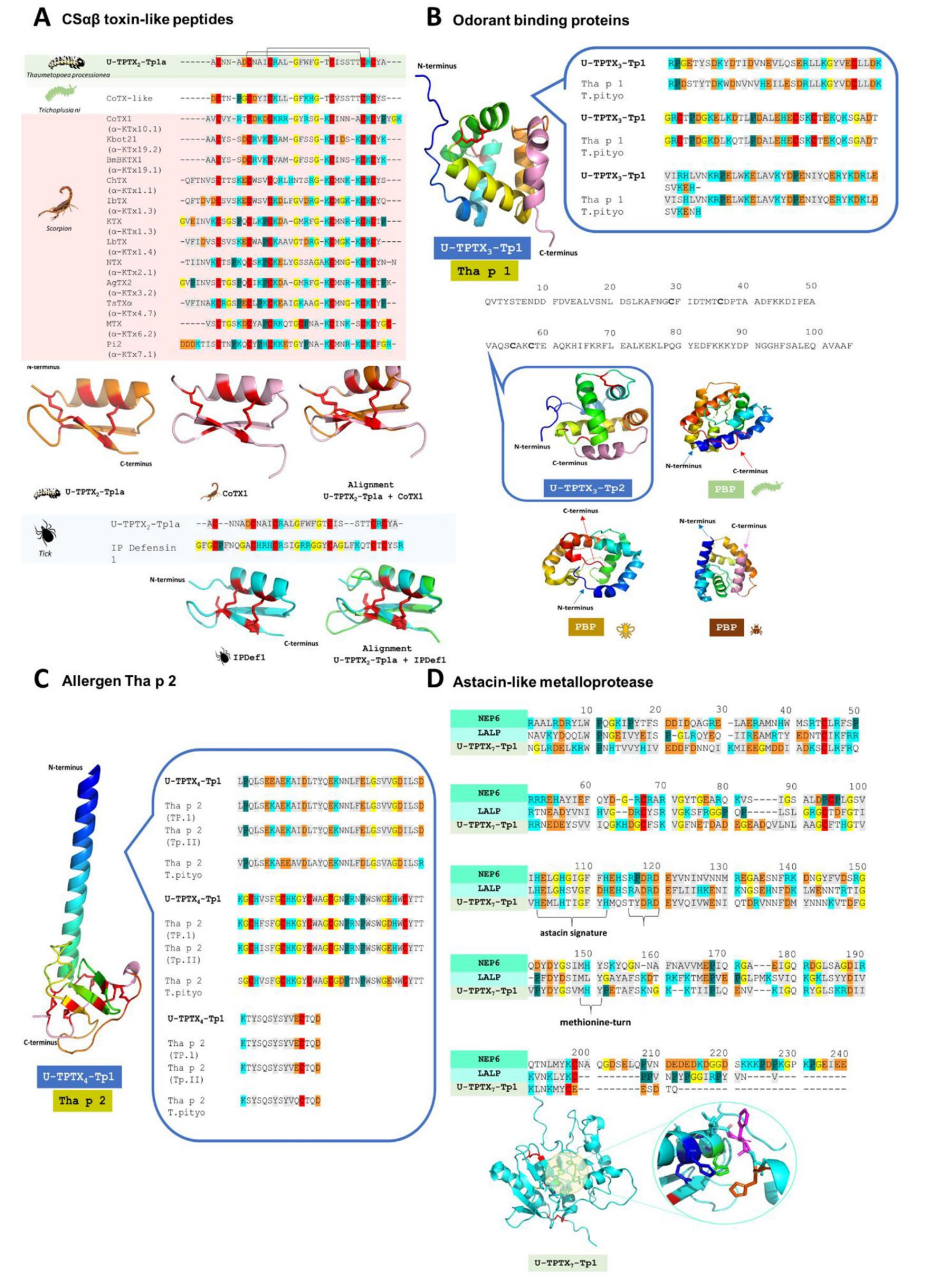
CSαβ toxin-like peptide family, odorant binding protein (OBP) family including allergen Tha p 1, allergen Tha p 2 family and astacin-like metalloprotease family in the venom of *Thaumetopoea processionea*. (**A**) CSαβ toxin-like peptide family: multiple sequence alignment of U-TPTX_2_-Tp1a of *T. processionea* with several scorpion toxins and with IP defensin 1 (IPDef1) from Ixodes persulcatus. 3D structure of U-TPTX_2_-Tp1a of *T. processionea*, 3D structure of cobatoxin-1 (CoTX1) from *Centruroides noxius* and 3D structure of IPDef1 predicted with AlphaFold2. 3D alignment of U-TPTX_2_-Tp1a with cobatoxin-1 and IPDef1. (**B**) The Odorant binding protein family: the 3D structure and sequence of U-TPTX_3_-Tp1 predicted by AlphaFold2 and visualized with PyMol. U-TPTX_3_-Tp1 was aligned with allergen Tha p 1 from *Thaumetopoea pityocampa*. The primary structure of another OBP, U-TPTX_3_-Tp2, featuring highlighted cysteine residues. The 3D structure of U-TPTX_3_-Tp2 was predicted with AlphaFold2 and visualized with PyMol. Three-dimensional structure of pheromone binding protein from *Bombyx mori* (pdb1dqe), *Apis mellifera* (pdb2h8v) and *Rhyparobia maderae* (pdb1org). (**C**) The allergen Tha p 2 family: 3D structure of U-TPTX_4_-Tp1 predicted with AlphaFold2 and visualized with PyMol. U-TPTX_4_-Tp1 was aligned with Tha p 2 from *Thaumetopoea pityocampa* and two Italian *Thaumetopoea processionea* species (Tp.I & Tp.II). (**D**) The astacin-like metalloprotease family: U-TPTX_7_-Tp1 of *T. processionea* is aligned via MAFFT with Loxosceles Astacin-Like Protease (LALP) from *Loxosceles intermedia* and Nematocyst expressed protein 6 (NEP-6) from *Nematostella vectensis*. The conserved astacin signature (HEXXHXXGFXH) or zinc-binding domain catalytic site and methionine-turn MXY are indicated. The three-dimensional structure of U-TPTX_7_-Tp1 is predicted with AlphaFold2 and visualized with PyMol. In this 3D structure, the zoom view represents the conserved astacin signature HEXXHXXGFXH. Residues in gray represent amino acids with a hydrophobic side chain, residues in light blue represent a positively charged chain, negatively charged side chain in orange, glycine in yellow, proline in green, cysteine in red, and amino acid with an uncharged polar side chain do not have a color

We identified three peptides (U-TPTX_2_-Tp1a, U-TPTX_2_-Tp1b, and U-TPTX_2_-Tp1c) featuring an identical mature peptide sequence but exhibiting subtle differences in their prepropeptide region (Supplementary Table S1). Structural analyses, conducted through sequence alignment of their mature peptide and 3D structure prediction using AlphaFold2, indicated that these peptides are part of an ancient family of disulfide-rich peptides characterized by a typical CSαβ scaffold (Fig. 3A). This scaffold consists of an alpha helix linked to an anti-parallel β-sheet, stabilized by specific disulfide bridges. In U-TPTX_2_-Tp1a, the disulfide bridges are formed by connecting the cysteines at C1-C4, C2-C5, and C3-C6. Via a Blastp search, 59.5% identity was found with a cobatoxin-like (CoTX) peptide from *Trichoplusia ni* (Insecta: Lepidoptera: Noctuidae) and 53.3% with Kbot21 from *Buthus occitanus tunetanus* (Arachnida: Scorpiones: Buthidae) [42]. The 3D structure predicted for U-TPTX_2_-Tp1a was aligned with CoTx1 from *Centruroides noxius* (Arachnida: Scorpiones: Buthidae) using PyMOL. The resulting RMSD value of 0.204 Å, calculated for 136 aligned atoms, signifies a notable degree of structural similarity between the predicted mature molecular structures of U-TPTX_2_-Tp1a and CoTX1. Subsequently, a multiple sequence alignment was conducted using known scorpion toxins with the typical scaffold that selectively modulates voltage-gated ion channels. The potassium channel activities are typically governed by a basic ring and a functional dyad [43]. For example, in CoTX1, this basic ring (R6, K10, R14, K28, and R18) interacts with Asp355 and Asp363 from the K_v_1.2 channel, and the functional dyad (K21 and Y30) obstructs K^+^ efflux and interacts with a hydrophobic cluster (Trp366, Trp367, and Tyr377) [44]. U-TPTX_2_-Tp1a lacks both the basic ring and functional dyad. Additionally, and important to share is that U-TPTX_2_-Tp1a shares a common feature with scorpion toxins: the short loop positioned between the first and second cysteines, often termed the ‘n-loop’ [45].

Furthermore, U-TPTX_2_-Tp1a exhibits similarity to Kbot21 and BmBKTxt, wherein the critical distinction lies in a single amino acid (D24 in Kbot21) on the functional surface [42]. This particular residue plays a decisive role in determining whether these toxins target K_v_ channels, like Kbot21 (blocks K_v_1.1, K_v_1.2, K_v_1.3, and K_v_1.6), or BK channels, as seen in BmBKTxt (exclusively on big and small conductance Ca^2+^-activated K^+^ channels without affecting K_v_ channels [46]. Notably, our analysis reveals a serine, not aspartic acid, glycine, or asparagine, at the critical position, unique among known scorpion toxins.

Next, we also found 43.75% similarity with a tick defensin, IP defensin 1 (IPDef1) from *Ixodes persulcatus* (Fig. 3A). With an RMSD value of 0.329 Å, calculated for 124 aligned atoms, it is suggested that despite their sequence differences, the two peptides show a close structural resemblance.

#### Odorant binding proteins

The presence and expression of a previously described odorant binding protein, Allergen Tha p 1 in *T. pityocampa*, was confirmed in the Belgian population of *T. processionea* venom (Fig. 3B). Through the UniProt Blastp database, we identified a 83.6% sequence identity between U-TPTX_3_-Tp1 and Tha p 1 from *T. pityocampa* complemented by a -10lgP score of 167.07 for U-TPTX_3_-Tp1. To the best of our knowledge, Tha p 1 was not described previously in *T. processionea*. The InterProScan results reconfirmed the classification of U-TPTX_3_-Tp1 within the OBP family, showcasing its characteristic six alpha helix structure with predicted cysteine bonds between C1-C2 and C3-C4.

Similar to U-TPTX_3_-Tp1, the presence of four other proteins with a predicted InterProScan domain of either insect odorant-binding protein A10/Ejaculatory bulb-specific protein 3 or pheromone/general odorant binding protein were confirmed in the extract of the setae of *T. processionea*. These OBPs belong to the short-chain soluble OBPs with approximately 100 residues. U-TPTX_3_-Tp2 and U-TPTX_3_-Tp3 show 70.3% similarity to the chemosensory protein 7 from *Bombyx mori*, U-TPTX_3_-Tp4 display 39.3% similarity to ejaculatory bulb-specific protein 3 from *Drosophila melanogaster* and U-TPTX_3_-Tp5 has 55.8% similarity to a general odorant-binding protein 28a-like. It is noteworthy that the five OBPs do not show the classic six conserved cysteine scaffolds [47]. According to their cysteine scaffold, they are categorized as minus-c OBPs due to the loss of cysteines [48, 49]. Moreover, the amino acid sequences of U-TPTX_3_-Tp(1–5) differ significantly from those described in the literature, such as OBP from *Heliothis virescens* (D2SNV4) or pheromone-binding protein (PBP) from *B. mori* (Q95VE9). Via AlphaFold2, it was predicted that U-TPTX_3_-Tp2 is a six α-helices-enriched globular protein with one cysteine bridge between C1 and C2. The overall 3D structure of U-TPTX_3_-Tp2 was compared with PBP from *B. mori*, *A. mellifera*, and *Rhyparobia maderae*, as shown in Fig. 3B. While the amino acid sequences of insect OBPs exhibit significant divergence between and within species, the overall structure of these OBPs remains highly conserved [50]. An actual difference is seen at the C-terminus. A medium length of the C-terminus from U-TPTX_3_-Tp2 closely approximates that of the PBP from *A. mellifera*.

#### Allergen Tha p 2

In addition to the major allergen Tha p 1, the other major allergen Tha p 2 was also identified in the venom of *T. processionea*. Through the UniProt Blastp database a 85.6% sequence overlap between U-TPTX_4_-Tp1 and Tha p 2 from *T. pityocampa* was found and complemented with a -10lgP score of 351.67. In contrast to U-TPTX_3_-Tp1 (allergen Tha p 1-like), InterProScan faces challenges in classifying U-TPTX_4_-Tp1 (allergen Tha p 2-like) into a family or in predicting the presence of certain domains. In Fig. 3C, the sequence alignment between U-TPTX_4_-Tp1 found in the Belgian population of *T. processionea*, Tha p 2 from the Italian population of *T. processionea* and Tha p 2 from *T. pityocampa* is visualized. Notably, at position 7, we observed a glutamic acid (E7) instead of a lysine (K7) [19]. The 3D structure of U-TPTX_4_-Tp1 was predicted via AlphaFold2 and visualized with PyMol (Fig. 3C). This structure is rich in serine, glycine and leucine and features a long alpha helix with a triple antiparallel β-sheet where the cysteine bonds are predicted between C1-C8, C2- C6, C3-C7, C4-C10, and C5-C9.

#### Enzymes putatively associated with toxicity

***Serine proteases***. For serine proteases, we identified with high confidence 20 proteins/peptides belonging to two families of serine proteases, namely trypsin-like S1A (U-TPTX_5_-Tp1-18) and PPAF-2-like CLIP domain (U-TPTX_6_-Tp1-2) (Supplementary Table S1). Interestingly, the typical S1 serine protease catalytic triad (His-Asp-Ser) was absent or disrupted by amino acid substitution in eight out of eighteen proteins/peptides (Supplementary Table S1) [51]. This alternation can be associated with a loss in proteolytic functionality, suggesting that S1 proteases in *T. processionea* may fulfill at least two distinct roles [52]. The PPAF-2-like containing serine protease family are recognized by a CLIP domain. This CLIP domain spans residues 63 to 109 in U-TPTX_6_-Tp1, and in U-TPTX_6_-Tp2, it ranges from residues 64 to 109 (Supplementary Table S1). Within this CLIP domain, a conserved tyrosine at position 103, characteristic of the PPAF-II family, is observed in both TPTX_6_-Tp1 and U-TPTX_6_-Tp2, contributing to their paper clip-like structural configuration [53]. Based on the identical arrangement of cysteine residues (3 disulfide bonds), this configuration has a domain architecture resembling that of β-defensins [54, 55]. It is important to note that the two proteins with a clip domain in the venom of *T. processionea* do not possess a catalytic domain.

***Metalloproteases***. Among the enzymatic proteins identified in the venom of *T. processionea* was also an astacin-like metalloprotease, U-TPTX_7_-Tp1. Via a multiple sequence alignment with Loxosceles Astacin-Like Protease (LALP) from *Loxosceles intermedia* (Arachnida: Araneae: Sicariidae) and Nematocyst Expressed Protein 6 (NEP-6) from *Nematostella vectensis* (Cnidaria: Anthozoa: Actiniaria), together with the inspection of the 3D predicted structure, several pieces of evidence support the idea that U-TPTX_7_-Tp1 is an astacin-like metalloprotease [56, 57]. Examining the three-dimensional arrangement, one can observe the presence of five-stranded β-sheets located at the N-terminal domain, accompanied by two alpha helices (Fig. 3D). This is followed by the active-site helix. This active-site helix includes parts of the astacin signature (HEXXHXXGFXH) found across astacins (Fig. 3D). More specifically, two histidine residues are involved in zinc coordination within the helix, and a glutamate residue serves as an acid for catalysis (proton donation of COOH side chain) [58]. Further on in the 3D structure, two more zinc ligands, the third histidine and a downstream tyrosine are present along with the methionine-turn MXY. This creates a hydrophobic base for the metal-binding site, playing a crucial role in maintaining the conformation of the metalloproteases (Fig. 3D) [59]. Next, an XXTXDRD motif was also found in U-TPTX_7_-Tp1. This domain slightly differs from the XXRXDRD domain described in the literature, where the charged residues establish interactions relevant to domain stability [58]. Further on, we identified four carboxypeptidase proteins in *T. processionea*. Carboxypeptidases are a subset of metalloproteases that belong to the M14 A/B family characterized by their ability to recognize a free C-terminal carboxyl group. U-TPTX_8_-Tp(1–4) belong to the carboxypeptidase M14A subfamily with the typical zinc ligand occurring within His-Ser/Ala-Arg-Glu-Trp/His and Xaa (hydrophobic residue)-His-Ser/Thr-Tyr-Ser-Gln/Glu motifs (Supplementary Table S1) [60]. Another subgroup of metalloproteases is the alanine aminopeptidase group. Analysis using InterProScan suggests five proteins, U-TPTX_9_-Tp(1–5), belonging to the zinc metallopeptidase family M1. Similar to metalloproteases, these aminopeptidases have a zinc-binding motif HEXXHX18E, with two histidine residues and the terminal glutamic acid that function as zinc ligands and catalytic site (Supplementary Table S1). Additionally, U-TPTX_9_-Tp(1–4) also displays a conserved GAMEN motif, whereas U-TPTX_9_-Tp5 has a GATEN motif. The amino acid prior to the GAMEN motif is suggested to be involved in the substrate specificity of the aminopeptidase. U-TPTX_9_-Tp(1–3) contains an alanine at this position which indicates a specificity for acidic residues. We have also found a serine residue at this position for U-TPTX_9_-Tp(4–5).

#### Enzyme inhibitors

**Fig. 4:**
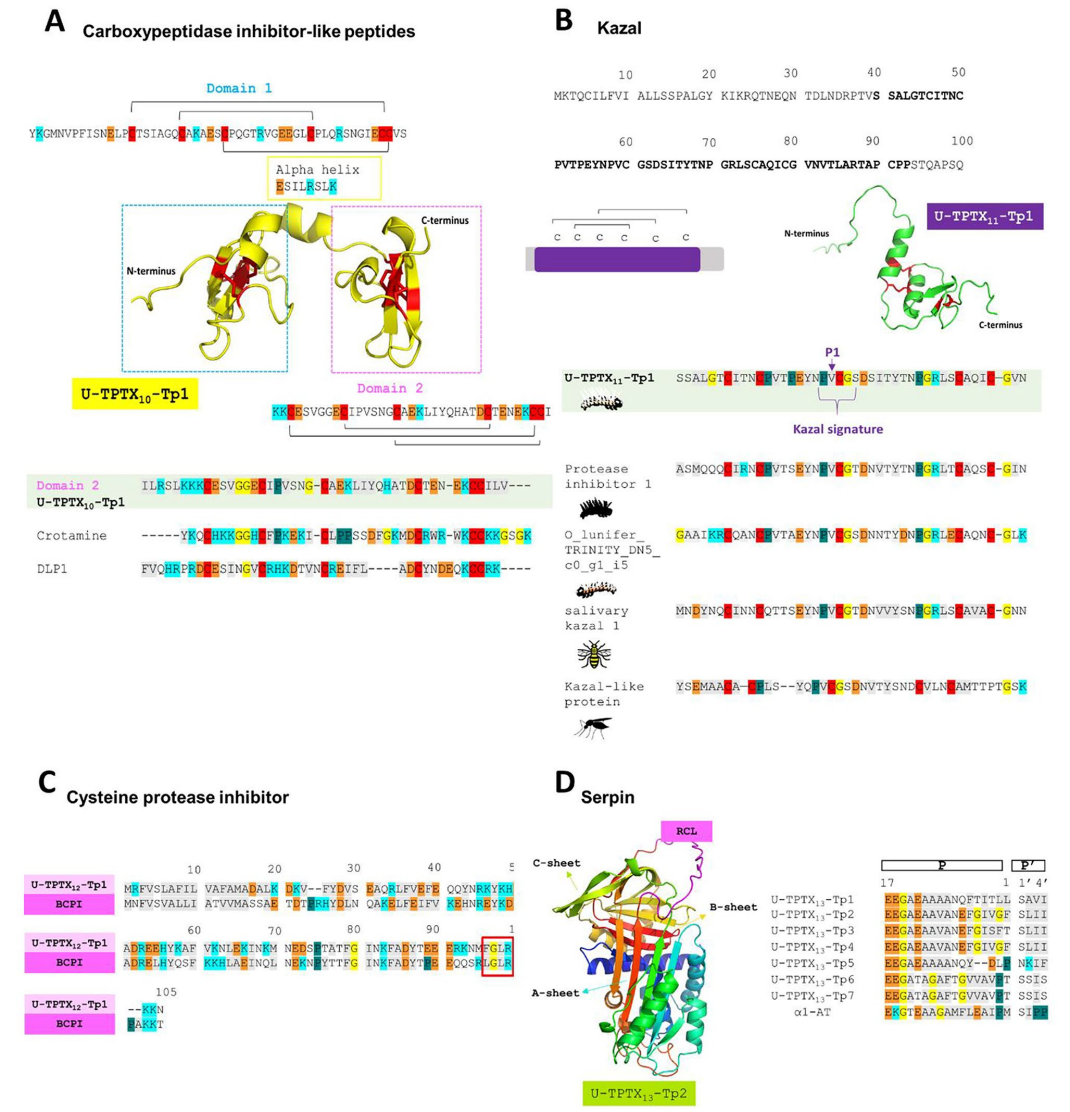
The enzyme inhibitors: the carboxypeptidase inhibitor-like peptide family, the Kazal family, the cysteine protease inhibitor family and the serpin family in the venom of *Thaumetopoea processionea.* (**A**) Carboxypeptidase inhibitor-like peptide family: 3D structure of U-TPTX_10_-Tp1 of *T. processionea* predicted with AlphaFold2 and visualized with PyMol. In blue and pink the two structurally identical domains. The sequence of Domain 2 aligned with crotamine from *Crotalus durissus terrificus* and Defensin-Like Peptide 1 (DLP1) from *Ornithorhynchus anatinus*. (**B**) The Kazal family: the primary structure of U-TPTX_11_-Tp1 with the Kazal domain in bolt. Six cysteine residues forming three disulfide bonds C1-C5, C2-C4 and C3-C6. 3D structure of U-TPTX_11_-Tp1 of *T. processionea *predicted with AlphaFold2 and visualized with PyMol. U-TPTX_11_-Tp1 was aligned with Kazal domain-containing proteins found in mosquitoes (*Culex tarsalis*, Kazal-like protein), wasps (*Nasonia vitripennis*, salivary Kazal 1) and caterpillars (*Lonomia obliqua*, protease inhibitor 1 and *Ochrogaster lunifer*, O_lunifer_TRINITY_DN5_c0_g1_i5). (**C**) The cysteine protease inhibitor family: U-TPTX_12_-Tp1 was aligned with the *Bombyx* cysteine protease inhibitor (BCPI). (**D**) The serpin family: 3D structure of U-TPTX_13_-Tp2 represents the highly conserved overall structure with A, B, and C-sheet and the Reactive Center Loop (RCL). U-TPTX_13_-Tp(1–8) was aligned with alpha-1-antitrypsin present in humans. Residues in gray represent amino acids with a hydrophobic side chain, residues in light blue represent a positively charged chain, negatively charged side chain in orange, glycine in yellow, proline in green, cysteine in red, and amino acid with an uncharged polar side chain do not have a color

***Carboxypeptidase inhibitor-like peptides.*** For U-TPTX_10_-Tp1, we obtained an intriguing hit via a Blastp search which unveiled a similar scaffold resembling a carboxypeptidase inhibitor observed in other Lepidoptera species (*Helicoverpa armigera* and *Bombyx mori*), ticks (*Rhipicephalus bursa*), and U-scoloptoxin19-Sm1a from the venom of the centipede *Scolopendra morsitans*. This scaffold features a double domain structure and the presence of 27 charged residues in the sequence (Fig. 4A). Each domain consists of a short α-helix followed by a small twisted antiparallel β-sheet, cross-linked by three disulfide bonds as shown in the 3D predicted structure of U-TPTX_10_-Tp1. Its distinctive two-domain nature prioritizes functionality over the optimization of conformational or folding properties [61]. Moreover, InterProScan revealed the presence of a myotoxin/anemone neurotoxin domain superfamily in the last segment or second domain of the sequence (amino acids 63–100) [62]. These myotoxins adopt a very similar fold to that of β-defensins, and as depicted in the multiple sequence alignment, U-TPTX_10_-Tp1 showed some conserved residues.

***Kazal***. Two proteins, U-TPTX_11_-Tp(1–2), were identified with a typical single or multiple Kazal domain architecture. This domain is named after the researcher Kazal, who first identified it in the pancreatic secretory trypsin inhibitor [63]. For U-TPTX_11_-Tp1, InterProScan analysis predicted the Kazal domain between positions 40 and 93 (Fig. 4B). In this Kazal domain a characteristic cysteine topology is visible, consisting of six cysteine residues forming three disulfide bonds C1-C5, C2-C4, and C3-C6 (Fig. 4B). These disulfide bonds help to stabilize the tertiary structure of the Kazal proteins, allowing them to maintain the specific fold and to interact with and inhibit serine proteases. Additionally, U-TPTX_11_-Tp1 contains a conserved (P1)CGxD Kazal signature. A reactive-site of inhibitor (P1) is located at the first position of this signature. The residue at this position exhibits a significant variability within the family. For instance, lysine or arginine at this position indicates trypsin inhibition, while an alanine, valine, or leucine is associated with elastase-like and subtilisin-like inhibition [64]. In U-TPTX_11_-Tp1, a valine is visualized in Fig. 4B. Via AlphaFold2, the typical Kazal three-dimensional structure of U-TPTX_11_-Tp1 is predicted. This 3D structure has two alpha helices and a short-stranded antiparallel β-sheet. To show the conserved region within the Kazal family, we performed a multiple sequence alignment of U-TPTX_11_-Tp1 with Kazal-like proteins described in other caterpillars (*O. lunifer* and *Lonomia obliqua*), a wasp (*Nasonia vitripennis*), and mosquito (*Culex tarsalis*) [65–68] (Fig. 4B). We examined the phylogenetic relationships of lepidopteran Kazal domain proteins, utilizing sequences obtained through a BLAST search of UniProtKB, the Kazal domain proteins identified in this study, and the Kazal domain proteins from *O. lunifer*. Our phylogenetic analysis shows that the node support for *O. lunifer* kazal (O_lunifer_TRINITY_DN5_c0_g1_i5) and *T. processionea* kazal (U-TPTX_11_-Tp1) is not very high but it suggests that they are orthologs (Supplementary Figure S3 and Supplementary Table S1). However, to conclusively determine orthology, a synteny analysis would be required.

***Cysteine protease inhibitor***. Besides Kazal domain protein inhibitors, two proteins with a cathepsin propeptide inhibitor domain were found. This domain is often produced as a component of a more extensive precursor protein (such as papain and cathepsin L) either at the N-terminus or as a prepropeptide. The release of this propeptide domain activates the protein. Alternatively, there are proteins exclusively comprised of single or multiple copies of this domain. Examples include the *Bombyx* cysteine protease inhibitor (BCPI), the *Drosophila* CTLA-2-like protein, and those found in Atlantic salmon (salarin) [69–71]. Since the latter proteins show homology to the pro-regions of cysteine proteases, they have been identified as cysteine protease inhibitors [72, 73]. U-TPTX_12_-Tp1 shows 88.89%, and U-TPTX_12_-Tp2 shows 78.03% sequence similarity with BCPI from *B. mori*. For the BCPI, it is known that the C-terminal (L96-G97-L98-R99) region is essential for the inhibition activity against the cathepsin L [69, 74]. These residues are comparable with the residues found in U-TPTX_12_-Tp1 and U-TPTX_12_-Tp2 (red box in Fig. 4C for U-TPTX_12_-Tp1).

***Serpins***. Serpins represent another class of protease inhibitor. In the venom of *T. processionea*, eight proteins, notably U-TPTX_13_-Tp(1–8), were identified with strong supporting evidence. The 3D predicted structure of U-TPTX_13_-Tp2 by AlphaFold2 shows the representative overall structure with a β-sheet A and the reactive center loop (RCL) commonly found in the serpin family (Fig. 4D) [41, 75]. Via the RCL, a serpin can bind to the active site of its target proteinase. More specifically, the RCL bears two crucial regions that govern the inhibitory function and specificity: the hinge region (residues P15-P9) and the P1 residue. A multiple sequence alignment of U-TPTX_13_-Tp(1–8) reveals mutations in the conserved hinge region compared to alpha-1-antitrypsin in humans (Fig. 4D). These mutations suggest a potential shift in the serpin’s function, indicating a tendency to act as a substrate rather than an inhibitor [76]. The conserved residues, spanning from P9 to P12, comprising short-chain amino acids (alanine) and a conserved glycine at P15, highlight specific structural elements. The residue at position P1 in the primary structure often hints at the target substrate. For instance, the literature indicates that, typically, a methionine (known as the Pittsburgh mutation) or arginine is found at this position [77]. The methionine mutation is associated with bleeding disorders, while arginine influences the coagulation cascade, affecting thrombin and Factor Xa [77]. However, the serpins detected in the venom of *T. processionea*, U-TPTX_13_-Tp(1–8), show variability at this position (threonine, proline, phenylalanine, or leucine). This diversity hints at U-TPTX_13_-Tp(1–8) potentially targeting multiple substrates.

#### Chitin-modifying enzymes, chitin-binding proteins and cuticle-proteins involved in the remodeling of the setae

**Fig. 5:**
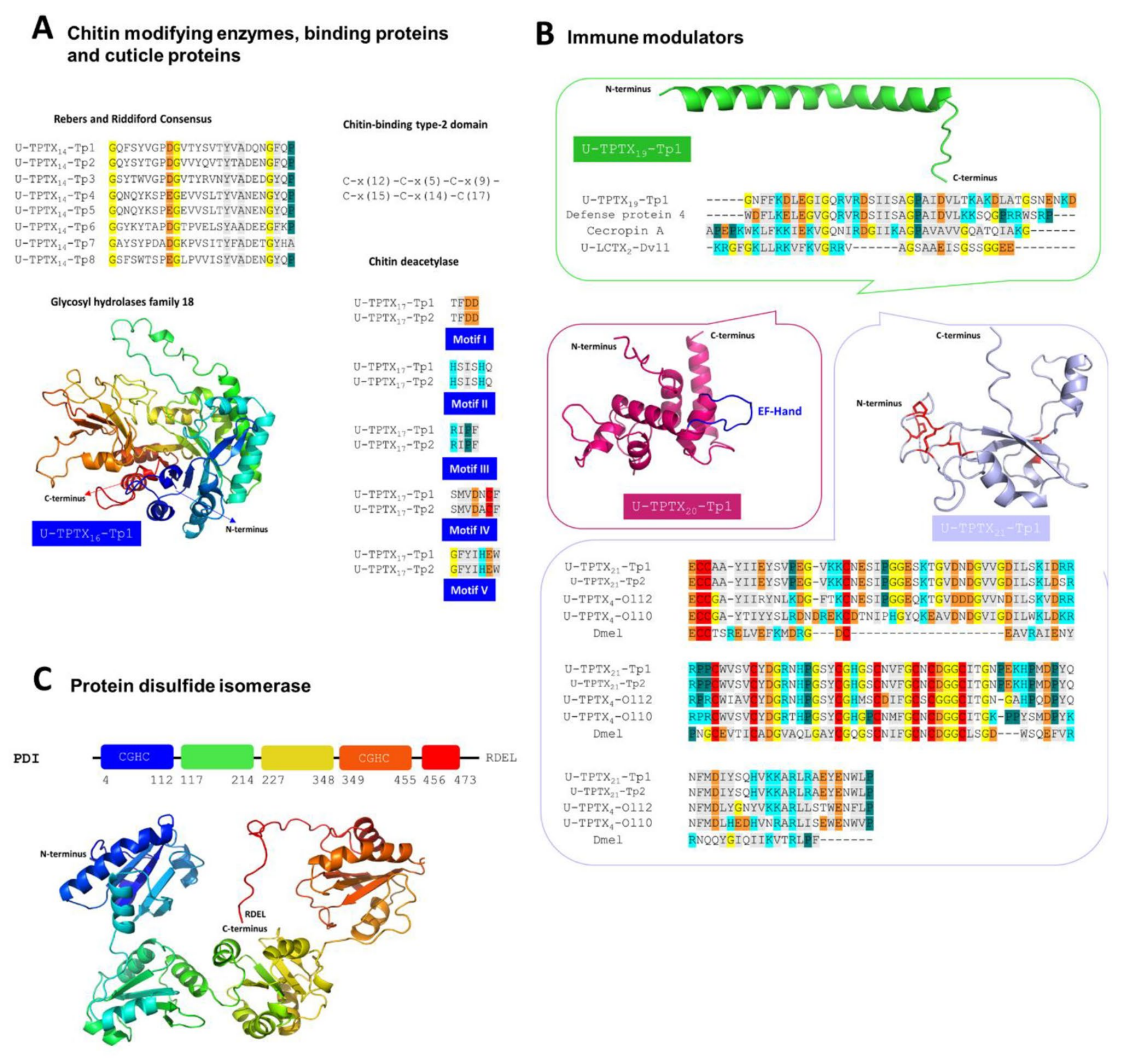
The chitin-modifying enzymes/chitin-binding proteins and cuticle-proteins, immune modulators, and protein disulfide isomerases in the venom of *Thaumetopoea processionea*. (**A**) The chitin-modifying enzymes/chitin-binding proteins and the cuticle-proteins: U-TPTX_14_-Tp(1–8), found in the setae extract of *T. processionea* with the Rebers and Riddiford Consensus (R&R Consensus). Three cuticle proteins, U-TPTX_15_-Tp(1–3), with a chitin-binding type-2 domain characterized by six cysteine in a C-x(12)-C-x(5)-C-x(9)-C-x(15)-C-x(14)-C(17) motif. The overall structure of U-TPTX_16_-Tp1 with the classic (β/α) TIM-barrel found in family 18 glycoside hydrolases. U-TPTX_17_-Tp1 and U-TPTX_17_-Tp2 with 5 characterizing motifs (motif I – V) similar to the chitin deacetylase 8 family. (**B**) The immune modulators: Green: cecropin. Multiple sequence alignment of U-TPTX_19_-Tp1 with cecropin A from Hyalophora cecropia and U-LCTX_2_-Dv11 from Doratifera vulnerans. Structure predicted by AlphaFold2. Pink: lysozyme. U-TPTX_20_-Tp1 structure predicted by AlphaFold2 with EF-hand domain colored in blue. Light purple: Diedel. U-TPTX_21_-Tp1 structure predicted by AlphaFold2. Multiple sequence alignment of U-TPTX_21_-Tp1 and U-TPTX_21_-Tp2 with U-TPTX_4_-Ol10 and U-TPTX_4_-Ol12 from *Ochrogaster lunifer* and Diedel from *Drosophila melanogaster* (Dmel). (**C**) The protein disulfide isomerase family: structure predicted by AlphaFold2 consists of five structure domains colored in blue, green, yellow, orange and red. The red box indicates the important residues for inhibitory activity in the C-terminus. Residues in gray represent amino acids with a hydrophobic side chain, residues in light blue represent a positively charged chain, negatively charged side chain in orange, glycine in yellow, proline in green, cysteine in red, and amino acid with an uncharged polar side chain do not have a color

The main building block of the setae from *T. processionea* is chitin. Therefore, it is not surprising that several proteins that can bind to chitin have been found in the venom extract of *T. processionea*. These chitin-binding proteins can be categorized into various distinct families based on the existence of specific amino acid sequence motifs [78, 79]. The majority of the chitin-binding proteins, U-TPTX_14_-Tp(1–8), have the Rebers and Riddiford domain (R&R domain) and belong to the CPR family [80]. The CPR family can be characterized by the motif G-x(7)-[DEN]-G-x(6)-[FY]-x-A-[DGN]-x(2,3)-G-[FY]-x-[AP] which is restricted to arthropods (Fig. 5A) [80]. Besides the CPR family, three proteins, U-TPTX_15_-Tp(1–3), were found with a chitin-binding type-2 domain. This domain is characterized by having six cysteine in a C-x(12)-C-x(5)-C-x(9)-C-x(15)-C-x(14)-C(17) motif (Fig. 5A). U-TPTX_15_-Tp2 and U-TPTX_15_-Tp3 bear a double chitin-binding type-2 domain. This double-domain structure may suggest that each molecule can bind two chitin fibers and thus form a three-dimensional chitin U-TPTX_15_-Tp(2/3) network [81]. Additionally, chitinases were found (Fig. 5A). These chitinases break down β-1,4-glycosidic bonds and thereby facilitate the degradation of chitin. U-TPTX_16_-Tp1, classified as a V chitinase with catalytic domains belonging to family 18 glycosyl hydrolases, displayed mutations in both motifs of the GH18 catalytic domain. These mutations involved alterations in motif I (KXXXXXGG(W-> D)) and motif II (FDGXDL(D-> A)W(E-> Q)(Y-> F)(P-> A)) (Supplementary Table S1) [82]. In Fig. 5A, the overall structure of U-TPTX_16_-Tp1 is shown. This structure comprises the classic (β/α)8 TIM-barrel (triosephosphate isomerase) found in family 18 glycoside hydrolases [83]. Besides chitinases of family 18, chitin deacetylases were also found. U-TPTX_17_-Tp1 and U-TPTX_17_-Tp2 display a 70% similarity with chitin deacetylase 8 of *B. mori*. Together with chitinases, chitin deacetylases play a crucial role in molting, developmental regulation, pathogenicity, immune defense, and remodeling of insect exoskeleton in *T. processionea* [5]. They are characterized by five motifs (motif I – V) that follow a typical pattern [84]. Motif I contains two aspartic acids, with the first serving as the catalytic base and the second acting as zinc-binding residues. Motif II contains two zinc-binding histidine residues. In motif III, an arginine is crucial for stabilizing the catalytic base of motif I. Notably, motif IV and motif V vary among the chitin deacetylase families described by Liu et al. (2019), contributing to a more open and wider active pocket [85]. Overall, it can be argued that U-TPTX_17_-Tp1 and U-TPTX_17_-Tp2 show conservation of the active sites, suggesting a shared catalytic mechanism among the chitin deacetylase. Finally, additional proteins, namely U-TPTX_18_-Tp(1–8), were identified, exhibiting similarities to other cuticle proteins found in caterpillars, contributing to their role in the formation and maintenance of the insect cuticle [5].

#### Immune modulators and antimicrobial-like proteins

Another category comprises peptides putatively associated with the immune system, serving as a defense mechanism against pathogens and parasites. *T. processionea* has incorporated a cecropin-like venom component, U-TPTX_19_-Tp1, from this peptide class. U-TPTX_19_-Tp1 was aligned with cecropin A from *Hyalophora cecropia* (Fig. 5B, **green**), enabling us to identify a signature represented by the pattern [KR]-[KRE]-[LI]-[ED]-[RKGH]-[IVMA]-[GV]-[QRK]-[NHQR]-[IVT]-[RK]-[DN]-[GAS]-[LIVSAT]-[LIVE]-[RKQS]-[ATGV]-[GALIV]-[PAG] [86]. An example of this signature is observed in U-TPTX_19_-Tp1, which exhibits the sequence K-D-L-E-G-I-G-Q-R-V-R-D-S-I-I-S-A-G-P (Fig. 5B, **green**). Moreover, U-TPTX_19_-Tp1 also exhibits similarities to defensin protein 4 from the deadly *Lonomia obliqua* and U-LCTX_2_-Dv11 from *Doratifera vulnerans* (Fig. 5B, **green**) [87]. As predicted by AlphaFold2, the structure of U-TPTX_19_-Tp1 exhibits one long α-helix and no two α-helices connected by a hinge region as reported for several other cecropins.

In addition to the cecropin family, *T. processionea* has recruited a lysozyme, U-TPTX_20_-Tp1, with 83.3% similarity with a lysozyme found in *Manduca sexta* (Fig. 5B, **pink**). Via InterProScan it was found that U-TPTX_20_-Tp1 has an EF-hand domain. This type of domain is found in many calcium-binding proteins and consists of a twelve-residue loop flanked on both sides by an α-helix [88]. In this loop, the 12th residue, aspartic acid, provides two oxygens for Ca^2+^ binding. The presence or binding of Ca^2+^ can enhance the structural stability of the lysozyme molecule. This structural stability is necessary to initiate an immune response by targeting the peptidoglycan of the Gram-positive bacterial cell wall, resulting in cell lysis [89].

Further on, two copies of Diedel-like proteins occur in *T. processionea*(Fig. 5B, **light purple)**. These toxins consist of 122 residues, including 10 cysteines and serve as a potential negative regulator of the JAK/STAT signaling pathway important for the innate immunity [90]. Via a multiple sequence alignment with Diedel-like protein found in *O. lunifer* (U-TPTX_4_-Ol12-10) and Diedel from *D. melanogaster* (Dmel), it became clear that U-TPTX_21_-Tp1 and U-TPTX_21_-Tp2 display a strong homology with the Diedel of *D. melanogaster* and *O. lunifer*. Also, the 10 cysteine residues seem to be conserved forming five disulfide bridges that attribute to the remarkable stability of the Diedel proteins. Compared to *D. melanogaster* Diedel protein (Dmel), the sole distinction lies in the fact that both *T. processionea* and *O. lunifer* Diedel-like proteins exhibits an insertion of 20 residues positioned between the third and fourth cysteine residues [14]. AlphaFold2 predicted that these 20 residues form an extra α-helix on the opposite side of the β-sheet as previously described by Walker et al. (2023) for the Diedel-like proteins in *O. lunifer*. As documented in the literature, these Diedel proteins showcase a dual architectural structure, one belonging to the ferredoxin family-like fold and the other displaying a distinctive fold not observed in any other protein in the Protein Data Bank.

#### Glycoside hydrolase

In addition to the glycoside hydrolase families 18 and 22, which were previously discussed in relation to chitinase and lysozyme, we found, with solid evidence, two members of the glycoside hydrolase families 1, U-TPTX_22_-Tp(1–2), and two members of the family 13, U-TPTX_23_-Tp(1–2) in the venom of *T. processionea*. As suggested by their names, these proteins are capable of hydrolyzing glycosidic bonds, either between carbohydrates or between a carbohydrate and a noncarbohydrate. Both U-TPTX_22_-Tp1 and U-TPTX_22_-Tp2 matched with the glycosyl hydrolase family 1 domain via InterProScan and showed 82.1% and 72.2% similarity to a myrosinase-like protein from *Manduca sexta* and *Spodoptera litura*, respectively. An alignment of U-TPTX_22_-Tp1 and U-TPTX_22_-Tp2 with Myrosinase 1 from *Brevicoryne brassicae* shows the conservation of all critical enzymatic site residues, suggesting that U-TPTX_22_-Tp1 and U-TPTX_22_-Tp2 are probably functional (Supplementary Table S1). U-TPTX_22_-Tp1 and U-TPTX_22_-Tp2 might be responsible for releasing toxic compounds, similar to certain insects mimicking a host plant’s defense mechanism [91, 92]. In these cases, when the body is damaged, volatile isothiocyanates are released through glucosinolate hydrolysis, exhibiting direct toxicity to natural enemies. While the presence of myrosinase is well-established in various plants, its function in caterpillars or other venomous animals remains largely unknown [91]. However, it raises intriguing questions about its potential role in the physiology of caterpillars. In addition to members of glycoside hydrolase family 1, proteins resembling α-amylases and belonging to glycoside hydrolase family 13 were identified in the venom of *T. processionea* [93]. The α-amylases within the glycoside hydrolase group may aid in insect sugar metabolism and are mainly found in the insect salivary gland, digestive tracts and the female venom gland of ectoparasitoid wasps [94]. However, the function of these α-amylases in the venom is not fully understood yet.

#### Protein disulfide isomerases

The transcriptome and proteomic data analysis also highlighted the presence of protein disulfide isomerase (PDI) in the venom of *T. processionea* (Fig. 5C). The PDI found in *T. processionea* venom demonstrated 87.7% similarity to PDI described for *Spodoptera litura* (Insecta: Lepidoptera: Noctuidae) and 78.4% with rat PDI. Deduced from its primary and tertiary structure as predicted by AlphaFold2, PDI from *T. processionea* consists of five structural domains (blue, green, yellow, orange, and red). The blue and yellow domains are the catalytic domains featuring a CGHC active site sequence. As described in the literature, the yellow domain functions in binding substrate proteins, and the orange domain bears the C-terminal ER retention signal (RDEL) [95]. The ER retention signal RDEL was also seen in PDI from *S. litura*, while KDEL is found in rats and humans [95]. This PDI is responsible for the precise formation of the disulfide bonds in the cysteine-rich peptides described above and supports the folding, which is necessary for structural stability and function [96]. This function is derived from its general folding pattern, reminiscent of thioredoxin and gives it the capacity to catalyze thiol-disulfide interchange reactions [96].

## Discussion

Lepidopterism poses a widespread problem in Europe, impacting thousands of individuals who experience discomfort from an itchy mosaic of red bumps. Different newspaper articles highlight that there is no single or simple answer to which active component(s) is responsible for the provoked health symptoms. It becomes even harder when there is only minimal information on a particular caterpillar species. While scientific literature covers *T. pityocampa* venom, the knowledge gap concerning other processionary caterpillars such as *T. processionea* poses a significant hurdle.

In light of the presented results, it becomes evident that the scope of allergens extends far beyond the initially identified ones (Thaumetopoein, Tha p 1, and Tha p 2). Our study reveals the presence of numerous additional odorant binding proteins similar to the previously described Tha p 1. Moreover, we also identified other components that may have toxic or allergenic effects, suggesting a more complex interplay in the envenomation process. This highlights the necessity for a more comprehensive analysis of the composition of the venom to fully understand the spectrum of factors influencing the observed health effect. In our hypothesis, we suggest a complex interplay among various venom components.

To begin, we delve into the functions of the allergens Tha p 1 and Tha p 2. In our investigation of *T. processionea* venom, we identified proteins akin to those found in the closely related species, including Tha p 1-like U-TPTX_3_-Tp1 protein and the Tha p 2-like U-TPTX_4_-Tp1 protein [10–12, 19]. The exact function of these allergens remains unclear, leading to investigations into their influence on allergic responses. Tha p 1, for instance, has been observed in various non-venomous caterpillars at different stages of development [18]. Since venomous properties arise in both *T. processionea* and *T. pityocampa* only during their third life stage, it remains uncertain whether this allergen acts as the primary cause of symptoms. This uncertainty suggests a potentially intricate role throughout the caterpillar life cycle. Nonetheless, the sensitization status of individuals may influence this dynamic. Repeated exposure to *T. processionea* can intensify reactions, potentially influenced by Tha p 1-induced IgE-dependent allergies, similar to the situation in which the allergen Tha p 1 was originally detected [12]. While in non-sensitized individuals, other components may induce an IgE-independent reaction. Unlike Tha p 1, Tha p 2 is expressed exclusively during the venomous stages of the caterpillar, occurring simultaneously with the formation of the urticating setae [9–11]. The exclusive presence of Tha p 2 in *Thaumetopoea* species, highlights its unique role within this genus [19].

Besides the odorant binding Tha p 1-like U-TPTX_3_-Tp1 protein, other OBPs (U-TPTX_3_-Tp2-5) were identified in the venom of *T. processionea*. Their compact structure and soluble nature, make them highly resistant to denaturing by organic solvents, high temperatures, and proteolysis [47]. Many studies have documented diverse expression patterns and functions of OBPs in insects and vertebrates, but overall, OBPs are known to bind and transport water-insoluble compounds through the sensillar lymph to receptive membranes [47]. This ligand-binding mechanism is strongly influenced by the length of the C-terminus [47, 97]. In *T. processionea*, all OBPs have a medium length C-terminus that resembles that of the C-terminus of the pheromone binding protein (PBP) from *A. mellifera.* With this medium-length C-terminus, it is believed that the entrance of the ligand-binding pocket is covered [47].

In the context of an immune reaction, it is also crucial to highlight the potential impact of chitin, a structural component discovered in the venom. This chitin is a linear and insoluble structural polysaccharide that serves a vital role in maintaining the rigid structure of various insect parts, such as the setae in *T. processionea* or the exoskeleton of many other insects [98]. Normally, touching these chitin exoskeletons doesn’t trigger itch or inflammation, suggesting that chitin might not be directly accountable for the symptoms observed. However, the interesting aspect arises when considering the ability of the harpoon-shaped setae to easily penetrate and break off in the skin. This penetration can initiate a cross-talk that activates immune cells crucial for the defense against foreign substances as described by Elieh Ali Komi et al. (2018) and Holm et al. (2014) [99, 100]. While humans lack chitin in their bodies, they still produce chitinases as part of the response to chitin-containing pathogens, such as fungi. When chitinases break down chitin into smaller fragments, they can bind to the toll-like receptor 2 or activate T-lymphocytes, initiating a cell-mediated immune response [100]. This mechanism was previously explored in the context of a closely related species, *Thaumetopoea pinivora* [99]. Maybe this slow breakdown of chitin in our body hints towards an explanation of the delayed onset or prolonged effects.

Diverging from the typical allergens and the chitin structure, the venom composition also introduces small peptides including secapin-like, cecropin-like, and CSαβ toxin-like peptides. These peptides are acknowledged for their functions in signaling and immunomodulation, contributing to defense against various invaders like bacteria, fungi, and pathogens. However, instead of solely focusing on their immunomodulatory roles, these peptides may also exhibit potential toxic effects, sparking curiosity for a deeper exploration into their interactions with the host system. For instance, secapin-1 showcases serine protease inhibitor-like activities with anti-fibrolytic, anti-elastolytic, and antimicrobial activities while secapin-2 manifests hyperalgesic and edematogenic effects without hemolytic activity, mast cell degranulation, or chemotaxis [101, 102]. The seven secapin-like peptides discovered in the venom of *T. processionea*, U-TPTX_1_-Tp1-7, exhibit similarity to both these secapins. Furthermore, we uncovered comparable peptide sequences in the transcriptome of *T. pityocampa*, whereas Walker et al. (2023) documented their presence in *O. lunifer* [14]. Besides *Thaumetopoea* species, secapins are also identified in various other animals, including Hymenoptera, Coleoptera, Diptera, and Hemiptera [14]. In many of these species, secapins are present in only a small proportion, and many lack toxin-defensive adaptations, as described by Walker et al. (2023) [14]. This mirrors the idea that these secapins may have a conserved role in normal physiology. At the same time, it also suggests that molecular adaptation may play a role in the evolution of caterpillar venom to make these toxins defensive, paralleling the evolutionary dynamics observed in snake venom phospholipase A_2_ (PLA_2_) [14, 87, 103, 104]. Our Blast search results hint at potential conservation and convergent evolution among certain insect species. However, the limitations of phylogenetic analysis urge caution in drawing conclusions regarding the evolutionary origin of the TPTX_1_ family.

Another noteworthy case involves the identification of cecropin-like peptide U-TPTX_19_-Tp1. Under normal physiological activities of insects, cecropin peptides demonstrate cytolytic, antimicrobial, antifungal, and antiparasitic activities, contributing to innate immunity [105, 106]. However, it was demonstrated that a cecropin-like peptide from the caterpillar *D. vulnerans* induces pain by creating pores in the cell membrane of mice [87]. This suggests that evolutionary adaptations can repurpose innate immunity peptides like cecropins, transforming them into defensive venom peptides with pain-inducing capabilities [87].

Apart from activities centered around pore formation, certain toxins are known to disrupt regular physiological functions, inducing sensations like pain, itch, and inflammation. This is achieved by altering voltage-gated and ligand-gated ion channels and receptors, serving to deter predators. In the venom of *T. processionea*, U-TPTX_2_-Tp1a-c exhibits a Csαβ-fold, a structural motif commonly found in various tissues with high abundance across both venomous (most scorpion-venom peptides) and non-venomous insects (insect innate immune molecules) [45, 107, 108]. Within this peptide family, there are diverse evolutionary pathways and functional adaptions, making it challenging to conclusively determine the activities of TPTX_2_-Tp1a-c. For instance, similar to TPTX_2_-Tp1a-c, several scorpion-venom peptides lack a functional dyad yet effectively modulate K_v_ channels, and the short n-loop suggests a potential loss of antimicrobial efficacy and acquisition of neurotoxic properties [43, 45, 109]. Furthermore, due to structural similarities with a tick pruritogen, IP defensin 1 (IPDef1), TPTX_2_-Tp1a-c, may also modulate the Mas-related GPRC11/X1 or the MRGPRB2/X2 and in this way causing itch and acute inflammation [110, 111].

To make sure that the venom achieves its intended effect by initiating the toxin activation/refinement and simultaneously contribute to the efficient distribution of the venom components throughout the host body, most venomous animals have various enzymes that facilitate these processes to enhance the overall impact [52, 112]. Among these enzymes identified in the venom of *T. processionea* are serine proteases and metalloproteases. The family of serine proteases was the second-largest group in the venom of *T. processionea*. The S1 proteases, U-TPTX_5_-Tp(1–18), are among the most globally recognized and well-described proteinases found in venomous animals that cleave peptide bonds after Arg or Lys, such as trypsin [113]. While this study don’t directly investigate the role of these serine proteases, it is important to underscore their vital role in diverse venom-related functions such as inflammation, initiation of pain and itch, blood coagulation, blood vessel relaxation, smooth muscle contraction, and suppression of immunity [52, 65, 114–116]. Of particular interest is their involvement in itch sensation. For example, serine proteases, such as those secreted by dermatophytes, activate protease-activated receptor 2 (PAR2), which is a G-protein-coupled receptor abundant in epidermal keratinocytes [117]. The activation triggers the release of itch mediators, including leukotriene B4, thromboxane A_2_ and nitric acid, which can be blocked by a serine protease inhibitor (nafamostat), a PAR2 antagonist (FSLLRY-NH2) or a 5-lipoxygenase inhibitor [117, 118]. Aside from the S1 proteases, the venom of *T. processionea* also contains PPAF-2-like CLIP domain containing serine proteases, exemplified by U-TPTX_6_-Tp1-2 [113]. These serine proteases are commonly associated with immune responses. Upon injury or infection, they trigger the activation of prophenoloxidase (proPo), leading to the production of melanin which encapsulate foreign invaders such as bacteria, fungi, and parasites [113].

The presence of both S1 proteases and CLIP domain proteins may indicate that the venom components are activated while in storage in the setae, when the setae break upon contact with the skin, or possibly both. If then the venom components needs to be spread throughout the body, metalloproteases take over [58]. These metalloproteases disrupt the extracellular matrix of the skin, facilitating toxin dispersion and access to molecular targets throughout the body [56]. In the venom of *T. processionea*, we found three families of metalloproteases including astacin-like metalloproteases (U-TPTX_7_-Tp1), caroboxypeptidases (U-TPTX_8_-Tp1-4) and alanine aminopeptidases (U-TPTX_9_-Tp1-5). For example, the astacin-like metalloprotease U-TPTX_7_-Tp1 may act as a molecular scissor upon reaching blood vessels, potentially causing symptoms like edema, increased capillary permeability, inflammation, and blister formation or just play a role in digestion [56]. The five aminopeptidases (U-TPTX_9_-Tp(1–5)) may have an impact on the extracellular matrix of the victim’s skin and in this way causing pain perception [119].

The fact that we found enzyme inhibitors (carboxypeptidase inhibitors (U-TPTX_10_-Tp1), Kazal (U-TPTX_11_-Tp1-2), cysteine protease inhibitors (U-TPTX_12_-Tp1-2), and serpins (U-TPTX_13_-Tp1-8) alongside the enzymes in the venom did not come as a surprise. There is often a delicate balance between these two components in venomous secretions. The enzyme inhibitors may control the actions of enzymes (such as inhibiting the premature activation or blood clotting) but also display some similarities to known neurotoxic relatives [56, 65–68, 120–122]. Such neurotoxic activity may be present in the carboxypeptidase inhibitor-like double-domain peptide, U-TPTX_10_-Tp1. This double domain structure is less explored in the literature compared to single-domain peptides. While one might anticipate that the dual domain is crucial for improving conformational or folding properties, this assumption is incorrect [61]. Instead, the unique two-domain nature places a greater emphasis on prioritizing functionality [61]. The first domain of U-TPTX_10_-Tp1 bears a resemblance to the β-defensin fold family identified in mammals [62, 123]. These β-defensins are essential elements in the innate immune system and function as both antimicrobial agents and chemokines [62, 123]. Due to their amphipathic nature, they can interact with bacterial membranes and in this way provide protection against microbial threats [62, 123]. The second domain identified belongs to a Myotoxin/Anemone neurotoxin domain superfamily. A well-known example of a myotoxin is crotamine, an α-myotoxin present in the venom of the rattlesnake *Crotalus durissus terrificus* [43]. These toxins are commonly linked to myonecrotic activity, disruptions in mitochondrial calcium homeostasis, and the induction of immune system responses [43, 124]. They achieve this by interfering with the functions of mast cells, macrophages, lymphocytes, and monocytes while demonstrating modulating effects on voltage-gated ion channels [43, 124].

While our study is the first to identify numerous proteins and peptides in *T. processionea* venom, some important future challenges remain to be addressed. Firstly, the functional roles of all these venom components remain speculative without experimental validation. To tackle this limitation, we want to emphasize the importance of in vitro and/or in vivo assays to confirm the biological activities and mechanism of action of these venom components. Secondly, we note that the study represents a snapshot of the venom composition at a single time point. At this moment, it is not known when or where the toxins are made. The setae are part of the exoskeleton and are regenerated with each moult, which means that the stage of the moulting cycle could significantly influence the toxin production. Therefore, while the TPM values indicate that a specific transcript is relatively highly expressed compared to other transcripts within a sample, it does not necessarily provide information about the functional significance of that transcript. Future longitudinal studies of toxin expression dynamics across larval life stages and under different environmental conditions are likely to provide a deeper understanding of how venom profiles vary over time and potentially identify putative ecological functions of its components. In regard to the secapin-like TPTX_1_ family, further studies on their evolution should test whether they originated through a possible duplication of a segment or domain from a growth factor receptor protein. Revisiting the mass spectrometry data from the study conducted by Berardi et al. (2017) could be beneficial, as their research utilized a minimum ORF size that might not have been conducive to detecting these peptides.

## Conclusion

To conclude, this proteotranscriptomic database has allowed us to write out a molecular storyline, providing clues about which venom components are present in the venom of *T. processionea*. On the one hand the venom is very similar to other animal venoms in the sense that they contain several convergently recruited components, but on the other hand, there are also aspects that set it apart from other venoms, such as the potential influence of the two allergens, the odorant binding proteins, chitin and the small peptides. This information has been elusive for many years. Now, it marks a pivotal milestone in unraveling the mysteries behind *T. processionea* envenomation. Not only does it provide a solid foundation for understanding the underlying mechanisms, it also sets a valuable benchmark for comparing with other caterpillar species. Our belief is that this dataset will play a key role in paving the way for the development of a targeted medicine to treat envenomation effectively.

**Tables**.

### Electronic supplementary material

Below is the link to the electronic supplementary material.

Supplementary Material 1

Supplementary Material 2

## Data Availability

The datasets generated during and/or analyzed during the current study are available from the corresponding author on reasonable request.
