# Supplementary material for: Exploring oak processionary caterpillar induced lepidopterism (Part 1): unveiling molecular insights through transcriptomics and proteomics
Source: Cell Mol Life Sci. 2024 Jul 27;81(1):311. doi: 10.1007/s00018-024-05330-z (PMC11335235; doi:10.1007/s00018-024-05330-z)
Supplement: Supplementary file 1 — Supplementary Material 1 [file 18_2024_5330_MOESM1_ESM.docx]

Exploring Oak Processionary Caterpillar Induced Lepidopterism (Part 1):

Unveiling Molecular Insights through Transcriptomics and Proteomics

**Andrea Seldeslachts**^1^, Marius F. Maurstad^2^, Jan Philip Øyen^2,#^, Eivind Andreas Baste Undheim^2^, Steve Peigneur^1*^, Jan Tytgat^1*^

*^1^Laboratory of Toxicology and Pharmacology, Department Pharmaceutical and Pharmacological Sciences, Catholic University of Leuven, Leuven, Vlaams-Brabant, Belgium*

*^2^Centre for Ecological and Evolutionary Synthesis, Department of Biosciences, The University of Oslo, Oslo, Norway*

*^#^Current address: Division of biotechnology and plant health & viruses, bacteria and nematodes in forestry, agriculture and horticulture, Norwegian Institute of Bioeconomy Research (NIBIO), Oslo, Norway*

*Correspondence: [jan.tytgat@kuleuven.be](mailto:jan.tytgat@kuleuven.be); [steve.peigneur@kuleuven.be](mailto:steve.peigneur@kuleuven.be)

Supplementary Material

# Supplementary Figures


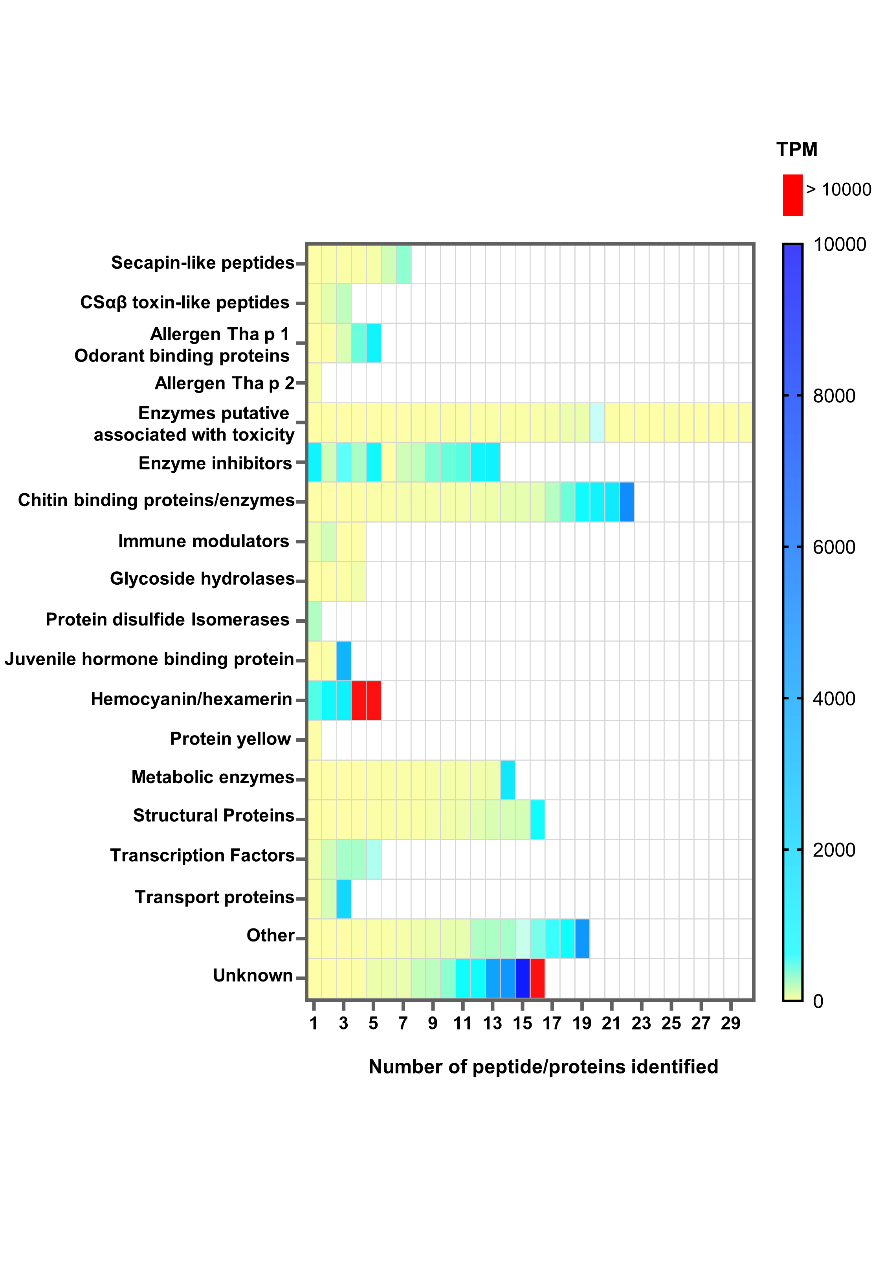


*Supplementary Fig. S1 RSEM-analysis of the venom categories of Thaumetopoea processionea. The heat map shows the 171 peptides/proteins with secretion signal sequences. The transcript per million (TPM) value is represented on a color scale where yellow indicates the lowest expression and dark blue signifies the highest expression. The red color shows the sequences with expression >1000 as shown on the right scale. The x-axis represents the number of peptides/proteins identified. To assess the relative abundance of each transcript, the paired trimmed reads were mapped back to the hybrid transcriptome assembly (generated from both Illumina NovaSeq and PacBio IsoSeq data) using bowtie2. The abundance values were calculated as Transcripts Per Million (TPM) from the mapped reads using RSEM v1.3.3*.


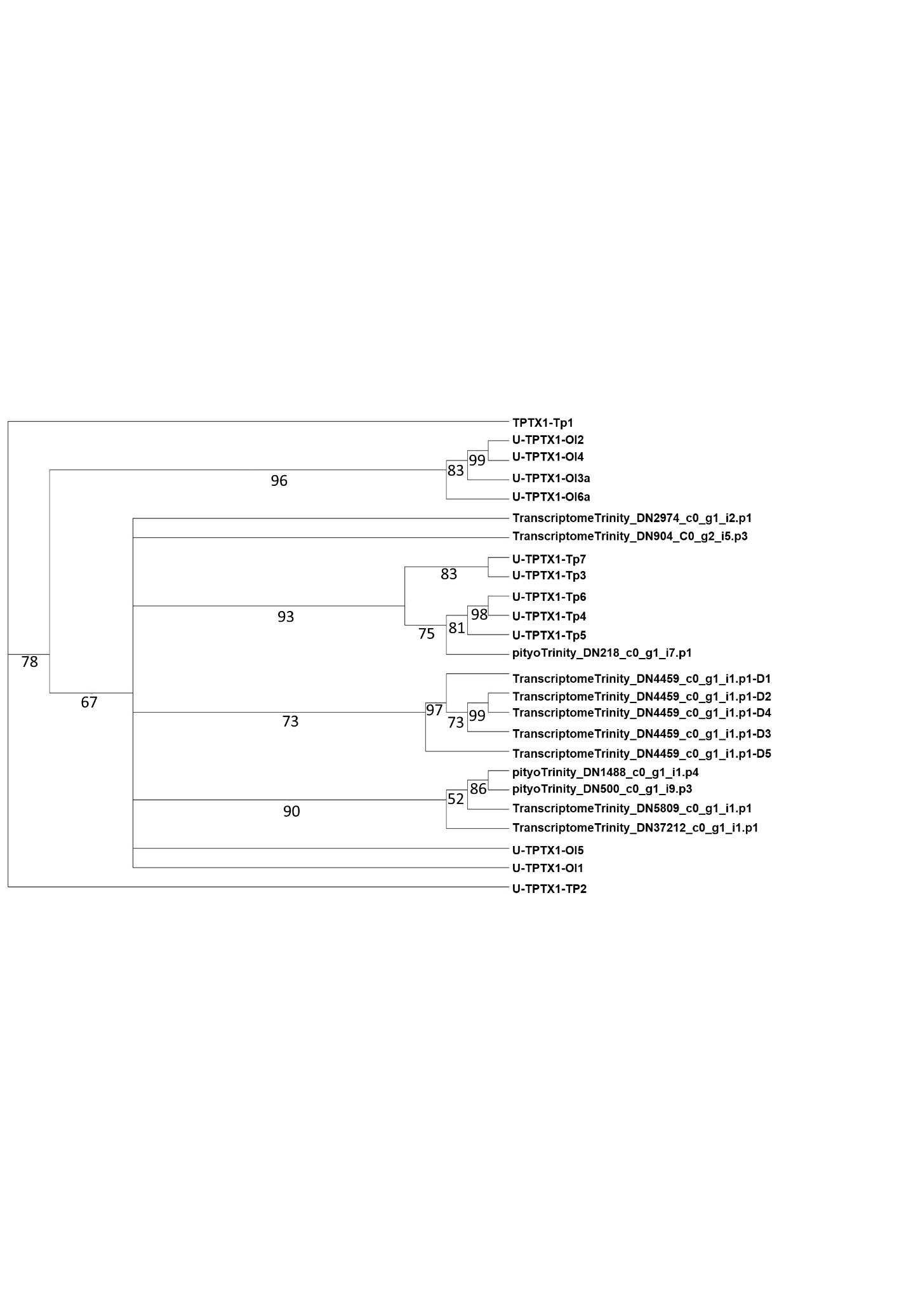


***Supplementary Fig. S2 Phylogenetic analysis of secapin-like sequences from Thaumatopoea processionea, Thaumetopoea pityocampa and Ochrogaster lunifer.*** *For the TPTX_1_ family analysis, the phylogenetic tree was constructed under the maximum likelihood VT+I+G4 model. This model was selected using Bayesian Information Criterion (BIC) to ensure the best fit for the data. Branch support values were estimated using ultrafast bootstrap, providing confidence assessments for the inferred evolutionary relationships. Bootstrap values, depicted as percentages (%), indicate the strength of support for each branch or node in the tree. Higher bootstrap values (>70%) suggest stronger confidence in the inferred evolutionary relationships, while lower values indicate greater uncertainty. Alignments were done with MAFFT v7 using L-INS-i and phylogenetic analysis were performed with IQTree v1.5.5 and Archaeopteryx 0.9928 beta.*


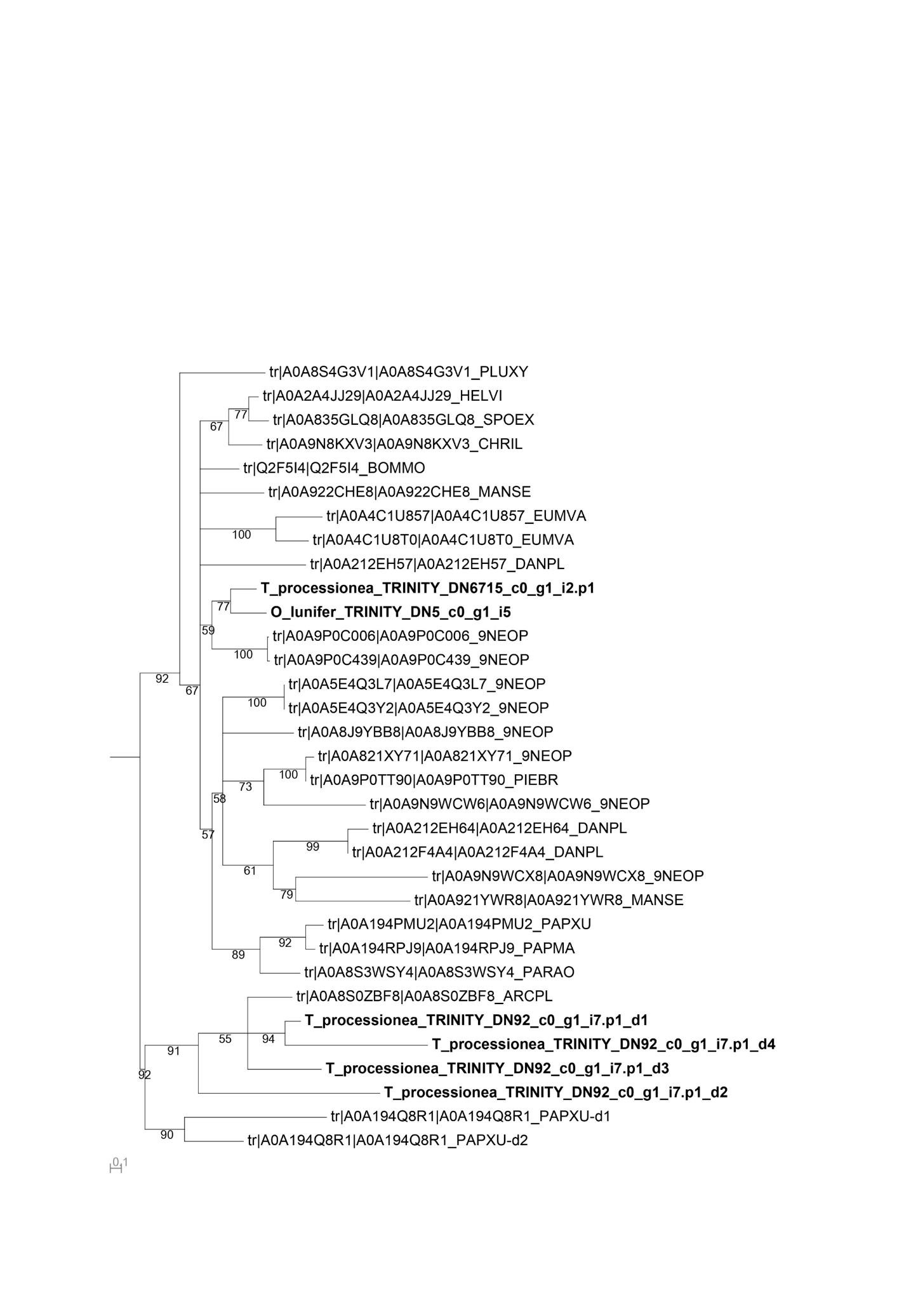


***Supplementary Fig. S3 Phylogenetic analysis of Kazal domain proteins from Thaumatopoea processionea and Ochrogaster lunifer.*** *For the Kazal family analysis, the alignments were performed with multi-domain peptides, which were split into their individual domains to ensure accurate alignment and tree construction. The phylogenetic tree was constructed under the maximum likelihood WAG+F+I+G4 model. This model was selected using Bayesian Information Criterion (BIC) to ensure the best fit for the data. Branch support values were estimated using ultrafast bootstrap, providing confidence assessments for the inferred evolutionary relationships. Bootstrap values, depicted as percentages (%), indicate the strength of support for each branch or node in the tree. Higher bootstrap values (>70%) suggest stronger confidence in the inferred evolutionary relationships, while lower values indicate greater uncertainty. Alignments were done with MAFFT v7 using L-INS-i and phylogenetic analysis were performed with IQTree v1.5.5 and Archaeopteryx 0.9928 beta.*
